# A meta-analysis of genome-wide association studies to identify candidate genes associated with feed efficiency traits in pigs

**DOI:** 10.1093/jas/skaf010

**Published:** 2025-01-23

**Authors:** Maria Rita Gonçalves da Silva, Renata Veroneze, Daniele B D Marques, Delvan A da Silva, Inaê I Machado, Luiz F Brito, Paulo S Lopes

**Affiliations:** Department of Animal Science, Federal University of Viçosa, Viçosa, MG, Brazil; Department of Animal Sciences, Purdue University, West Lafayette, IN 47907, USA; Department of Animal Science, Federal University of Viçosa, Viçosa, MG, Brazil; Department of Animal Science, Federal University of Viçosa, Viçosa, MG, Brazil; Department of Animal Science, Federal University of Viçosa, Viçosa, MG, Brazil; Department of Animal Science, Federal University of Viçosa, Viçosa, MG, Brazil; Department of Animal Sciences, Purdue University, West Lafayette, IN 47907, USA; Department of Animal Science, Federal University of Viçosa, Viçosa, MG, Brazil

**Keywords:** feed conversion ratio, genetic markers, genome-wide association studies, meta-analysis, residual feed intake

## Abstract

Pig production is an agricultural sector of great economic and social relevance to Brazil and global markets. Feed efficiency traits directly influence the sustainability of pig production due to the economic impact of feed costs on the production system and the environmental footprint of the industry. Therefore, breeding for improved feed efficiency has been a target of worldwide pig breeding programs. Genome-wide association studies (**GWAS**) enable the assessment of the genetic background of complex traits, which contributes to a better understanding of the biological mechanisms regulating their phenotypic expression. In this context, the primary objective of this study was to identify and validate genomic regions and candidate genes associated with feed conversion ratio (**FCR**) and residual feed intake (**RFI**) in pigs based on a comprehensive systematic review and meta-analysis of GWAS. The METAL software was used to implement the meta-analysis and the Bonferroni multiple testing correction considering a significance threshold 0.05. The significant single nucleotide polymorphisms (**SNPs**) in the meta-analysis were used to identify candidate genes, followed by a functional genomic enrichment analysis. The systematic review identified 13 studies, of which 7 evaluated FCR, 3 evaluated RFI, and 3 studies investigated both traits, with 160 and 96 SNPs identified for FCR and RFI, respectively. After the meta-analysis, 145 markers were significantly associated with FCR and 90 with RFI. The gene annotation process resulted in 105 and 114 genes for FCR and RFI, respectively. The enrichment analysis for FCR resulted in 16 significant gene ontology (**GO**) terms, while 6 terms were identified for RFI. The main GO terms were actin cytoskeleton (GO_BP:0030036), membrane (GO_CC:0016020), integral components of the peroxisomal membrane (GO_CC:0005779), and carbohydrate-binding (GO_MF:0030246). The main candidate genes identified were *MED18, PHACTR4, ABCC2, TRHDE, FRS2, FAR2* and *FIS1* for FCR, and ADGRL2, ASGR1, ASGR2, and MAN2B1 for RFI. These findings contribute to a better understanding of the genetic mechanisms associated with feed efficiency traits in pigs, providing a foundation for future improvements in pig breeding programs.

## Teaser

We identified various candidate genes associated with feed efficiency traits in pigs based on the integration of multiple studies, providing valuable information on the genetic mechanisms regulating these important traits.

## Graphical Abstract

**Graphical Abstract:**
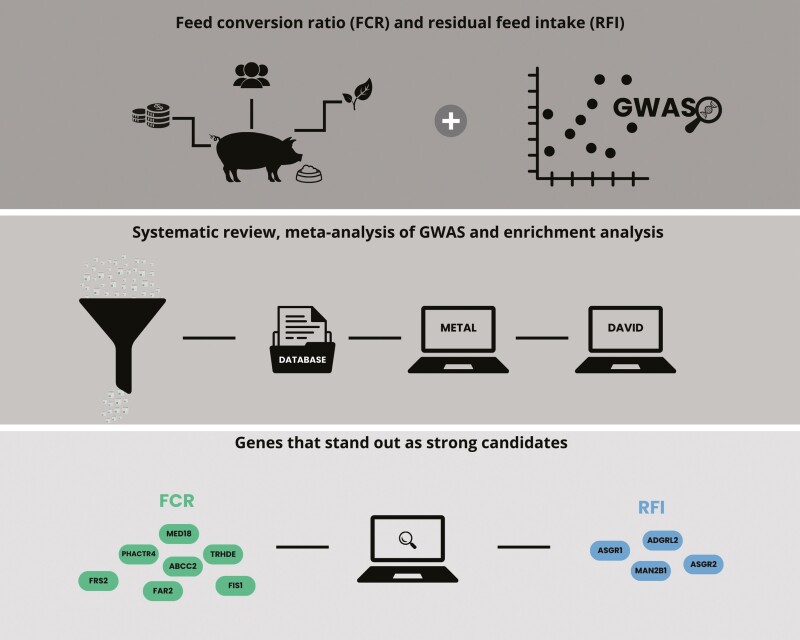

## Introduction

Feed efficiency traits are key breeding goals in livestock breeding programs due to their impact on production costs and the environmental footprints of animal production (Do et al., 2013; Brito et al., 2020; Pomar et al., 2021). The development of multiple dense single nucleotide polymorphism (**SNP**) panels, coupled with the significant reduction in genotyping costs in recent years, have driven the growing application of genome-wide association studies (**GWAS**) in livestock. GWAS focus on identifying genetic variants and quantitative trait loci (**QTL**) associated with relevant traits, such as feed efficiency. Therefore, GWAS contribute to a better understanding of the genetic mechanisms underlying phenotypic variability in the traits under study (Kronenberg, 2008; Magalhães et al., 2016).

Various GWAS have reported SNP associations and candidate genes for feed efficiency traits in pigs (Sahana et al., 2013; Ding et al., 2017, 2018; Miao et al., 2021; Li et al., 2022). However, there are significant differences in their methods, datasets, and results. For example, evaluating feed conversion ratio (**FCR**) in pigs, Sahana et al. (2013) identified significant SNPs located only on chromosome 14 (SSC14; *Sus scrofa* 14), while Miao et al. (2021) and Ding et al. (2017), who also evaluating FCR, identified significant SNPs located on SSC5 and SSC12, respectively. These differences were also observed in GWAS for residual feed intake (**RFI**) in pigs. Ding et al. (2018) reported significant SNPs located on SSC1 and SSC7, while Li et al. (2022) reported significant SNPs on SSC2, SSC8, and SSC15. These discrepancies among studies can be explained by the polygenic nature of these traits, population differences, SNPs included in the analyses, statistical methods used, and the sample sizes of the studies (Korte and Farlow, 2013; Fu et al., 2020). Therefore, methods for integrating results from different studies can leverage complementary information and improve the identification of QTL and candidate genes. In this context, meta-analysis is one of the main approaches used for integration of results from different studies to provide summarized outcomes, including genomic regions associated with phenotypes of interest (Evangelou and Ioannidis, 2013; Oliveira et al., 2024).

Meta-analysis of GWAS results can increase the power of detecting association signals and relevant genomic regions as compared to studies using individual datasets (Zeggini and Ioannidis, 2009). This is particularly relevant since single studies can be limited in terms of statistical power and the reliability of results when small datasets are used (Taherkhani et al., 2022). Thus, meta-analysis is becoming increasingly important in summarizing the results of GWAS for quantitative traits (De Bakker et al., 2008). In addition, meta-analysis of GWAS can identify potential false positive associations and highlight the more relevant genomic regions (Duarte et al., 2019). In this context, the primary objectives of this study were to identify and validate candidate genes associated with FCR and RFI in pigs based on a systematic review and meta-analysis of GWAS for these traits.

## Material and Methods

### Systematic review

A systematic review was performed to gather results from studies reporting associations between SNPs with FCR and RFI traits in pigs. The searches were carried out on the Web of Science (200,000 papers), Scopus (74 papers), and PubMed (8,806 papers) websites between June 26 and June 28, 2024. The search terms used were combinations of keywords with the following criteria: 1) species (“pig”, “swine”); 2) type of association test (“genome wide association”, “genome-wide association”, “GWAS”); and 3) term related to the trait evaluated (“feed efficiency”, “feed conversion”, “residual feed intake”, “RFI”). We also used the Boolean Operators “and” and “or”, which are conjunctions used to form combinations of keywords and improve the results obtained (Koutsos et al., 2019). Illustrative schemes of the searches performed are presented in Figures S1, S2, and S3. We used 2 combinations of keywords as presented below:

- “pig” **OR** “swine” **AND** “genome wide association” **OR** “GWAS” **AND** “feed efficiency” **OR** “feed conversion”
- “pig” **OR** “swine” **AND** “genome wide association” **OR** “GWAS” **AND** “feed efficiency” **OR** “residual feed intake” **OR** “RFI”

The search was conducted by 2 independent researchers. After performing the searches, the publications identified were added to the End Note application for subsequent steps. First, duplicate papers were excluded, 62,453, and then the query terms for each combination were checked to see if they appeared in the title and abstract of each paper. Thirty-two papers were selected after evaluating the titles, and 20 of these were selected after reading the abstracts. After this stage, these 20 studies were checked individually to analyze if the full text was available, if the species was a pig, if the methodology used was GWAS for FCR and RFI, and if the SNP, its genomic coordinate (chromosome and location on the chromosome) and its *P*-value were provided.

**Figure 1.**
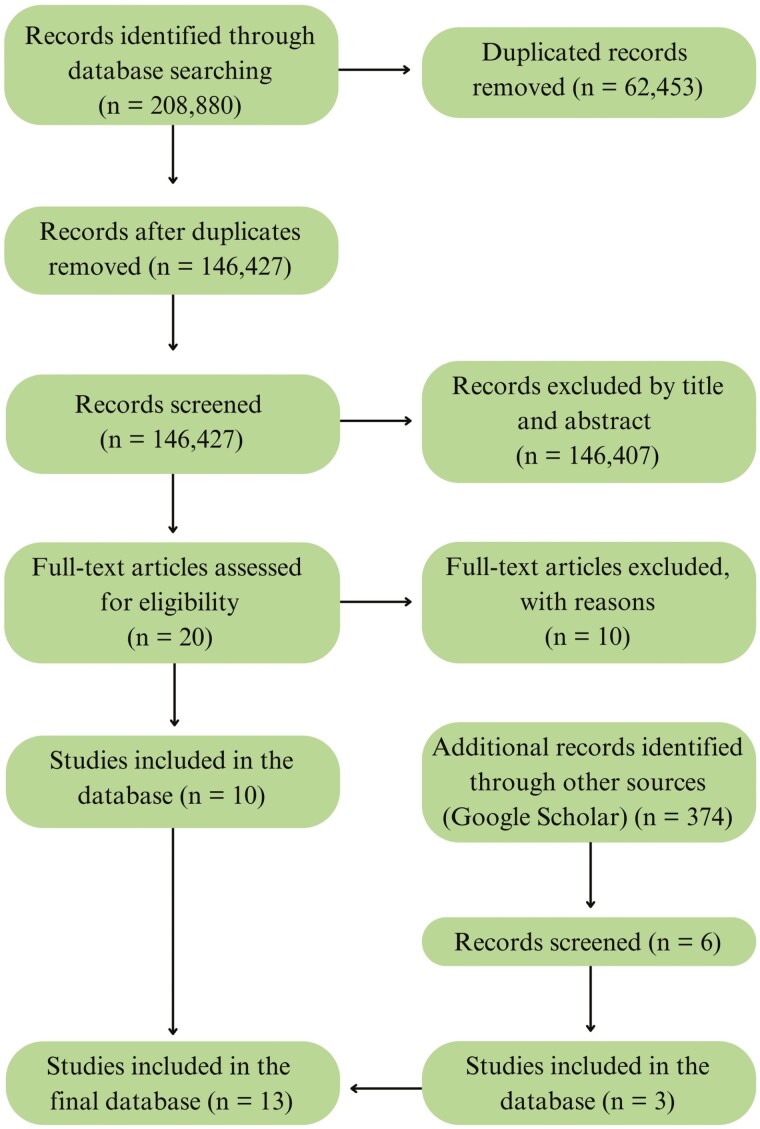
Flowchart of the papers selection process in the systematic review. The flowchart shows the stages of papers selection, starting with 208,880 identified records. After the complete filtering process, 13 studies were included in the final analyses.

After a thorough reading, 10 papers with insufficient information were excluded from further analyses. The 10 papers considered eligible for the research, after reviewing the full text, were included in the final set of studies to assemble the database. The following information was extracted from each paper: author, SNP chip panel information, traits, number of observations, significant SNPs identified, the chromosome and genomic position of the significant SNPs, and the associated *P*-values. An additional search was performed using Google Scholar to find papers that had not yet been selected. The same keyword combinations and article filtering process were used. This step resulted in 374 papers being found, from which 6 papers were selected according to the same criteria used in the full-text analysis. After this analysis, 3 studies were included in the set of studies to make up the database. Therefore, 13 studies were used to assemble the final database, of which 10 and 6 studies evaluated FCR and RFI, respectively. Three papers reported results for both traits (Ding et al., 2018; Fu et al., 2020; Li et al., 2022). The paper selection process is summarized in Figure 1.

### Meta-analysis

The genomic location of each SNP was updated to the most recent reference genome (*Sus scrofa* 11.1) using the Biomart tool available on the Ensembl website. After the filtering process (as detailed in the Systematic Review section), which removed duplicate articles, keywords and articles were carefully reviewed and 160 SNPs for FCR and 96 for RFI were included in the final database. The meta-analysis was based on the weighted *Z* scores model, implemented in the METAL software (Willer et al., 2010), which is a tool for meta-analysis genome-wide association scans. This method considers the *P*-value associated with the SNP, direction of effect, and the number of individuals included in each GWAS study. Firstly, for each SNP, a reference allele was selected and a *z*-statistic characterizing the evidence of association was calculated. The *z*-statistic summarizes the magnitude and the direction of the effect relative to the reference allele and all studies were aligned to the same reference allele. Next, an overall z-statistic and *P*-value were then calculated from a weighted sum of the individual statistics. Weights are proportional to the square-root of the number of individuals examined in each sample and selected, such that the squared weights sum to 1.0 (Sanna et al., 2008; Willer et al., 2010). However, in this study, the direction of the effect was considered to be the same for all the markers, since the reference allele was not available in the selected papers. After the meta-analysis, to establish statistically significant associations, the Bonferroni correction was applied considering an alpha (α) = 0.05 for the genome-wide significance level, dividing α by the number of SNPs tested in the meta-analysis.

### Gene annotation and functional analyses

The significant SNPs obtained from the meta-analysis were used to identify candidate genes associated with FCR and RFI. The SNP—gene annotation was carried out using a window of 32 kb on either side of each SNP. We performed gene annotation using the Biomart R package (Durinck et al., 2005), following Verardo et al. (2016). Gene annotations, including gene identifiers, gene symbols, and locations were retrieved from the databases. Functional enrichment analyses were then conducted using the Database for Annotation, Visualization, and Integrated Discovery (DAVID) (Huang et al., 2009; Sherman et al., 2022). The analyses included gene ontology (**GO**) and KEGG pathways to identify biological processes, molecular functions, cellular components, and biological pathways associated with the candidate genes identified after the meta-analysis. A *P*-value < 0.05 was considered statistically significant.

### Gene network analysis

A gene network analysis was carried out using the GeneMANIA tool (Warde-Farley et al., 2010) as a plug-in for Cytoscape (Shannon et al., 2003), with the human gene annotation as a reference. These analyses aimed to investigate the interactions between genes related to FCR and RFI, since this knowledge is fundamental to the understanding of the biological mechanisms underlying the phenotypic variability of the target traits. This is a tool to search for integration between genes or sets of genes and allows the evaluation of co-expression, physical integration, genetic integrations, shared protein domains, and co-localization. This analysis was performed with the genes identified based on the meta-analysis and only genes with complete identification and symbol information were used to build the gene networks. Three gene networks were built: one for the genes associated with FCR, one for the genes associated with RFI, and one combining the genes identified for both traits.

## Results

### Systematic review

The systematic review included 13 articles investigating feed efficiency traits in pigs, such as FCR and RFI. The studies varied in terms of breeds used, sample sizes, and methodological approaches. The oldest publication was from 2013 (Onteru et al., 2013; Sahana et al., 2013), and the most recent was from 2022 (Li et al., 2022), highlighting the ongoing interest in complex feed efficiency traits over time. The study with the smallest sample size had 217 animals (Bai et al., 2017), while the largest sample size was 3,672 animals (Miao et al., 2021). Furthermore, the main breeds studied were Duroc, Yorkshire, Landrace, and a predominantly Pietrain mixed line (Maxgro).

Belous et al. (2019) evaluated FCR in Duroc pigs, based on total feed intake relative to weight gain during the growth period. A total of 715 animals were used, genotyped with the Illumina Porcine SNP60 BeadChip, covering 44,810 SNPs. The significance threshold was based on the Bonferroni method (*P* < 1.12 × 10⁻⁶). Ding et al. (2017) also investigated FCR in Duroc pigs, with 338 animals genotyped using the same SNP chip, including 35,791 SNPs after the quality control. The significance criterion was adjusted with the Bonferroni correction, with an adjusted *P*-value of 1.4 × 10⁻⁶.

Fu et al. (2020) analyzed both FCR and RFI in Landrace pigs, using 296 animals genotyped with the PorcineSNP50 BeadChip. This study used 41,272 SNPs and applied the FDR correction method for significance thresholds.

Horodyska et al. (2017) evaluated FCR in a predominantly Pietrain terminal line (Maxgro boars), using 952 animals and the PorcineSNP60 BeadChip (48,440 SNPs). Phenotypes were used as EBVs, and Bonferroni correction was applied (− log10[p] = 6).

Li et al. (2022) investigated FCR and RFI in Duroc and Yorkshire pigs, using 880 animals for RFI and 485 for FCR. The CAU50K chip was used, covering 32,322 SNPs for Yorkshire and 25,539 for Duroc. Phenotypes were used as dEBVs, and Bonferroni correction was applied to the significance threshold.

Miao et al. (2021) evaluated FCR in Yorkshire pigs, with 3,672 animals genotyped using the Illumina PorcineSNP60 BeadChip, covering 31,236 SNPs. Phenotypes were also used as dEBVs, and the significance threshold was adjusted to − log10(p) = 5.796.

Reyer et al. (2017) studied FCR in Maxgro pigs, analyzing 846 animals genotyped with the Porcine SNP60 BeadChip (51,509 SNPs). FCR was calculated as the ratio of daily feed intake to body weight gain. The significance was defined as *P* < 2.19 × 10⁻⁶.

Sahana et al. (2013) analyzed FCR in Duroc pigs (3,071 animals) using the Illumina Porcine SNP60 BeadChip (30,847 SNPs). Phenotypes were used as EBVs, and Bonferroni correction was applied to the significance threshold.

Wang et al. (2015) investigated FCR in 796 Duroc pigs genotyped with the Illumina Porcine SNP60 BeadChip. FCR was measured within the weight range of 30 to 100 kg, with an adjusted *P*-value of less than 0.05 as the significance criterion.

Bai et al. (2017) focused on RFI using 217 pigs genotyped with the Illumina PorcineSNP60v2 BeadChip (50,544 SNPs). The Bonferroni correction method was applied, with *P* < 9.89 × 10^−7^.

Ding et al. (2018) evaluated FCR and RFI in Duroc pigs (1,008 animals) genotyped with the Geneseek Porcine 50K SNP Chip (32,446 SNPs). Bonferroni correction was applied, with *P* < 3.08 × 10^−5^.

Do et al. (2014) analyzed RFI in Yorkshire pigs (596 animals), using the PorcineSNP60 Illumina chip (37,192 SNPs). Phenotypes were used as dEBVs, and the significance threshold was adjusted with the Bonferroni correction for the number of independent tests (*P* = 1 × 10^−4^).

Onteru et al. (2013) also evaluated RFI in Yorkshire pigs, with 1,410 animals genotyped using the Illumina PorcineSNP60 BeadChip (50,953 SNPs).

To assemble the database, the following information was extracted from each paper: author, SNP chip panel, trait(s), number of observations, significant SNP, the chromosome and genomic position of the significant SNP, and the associated *P*-value. An overview of the papers is presented in Table 1. The complete dataset collected is presented in Table S1.

**Table 1.** Description of the papers selected in the systematic review

Paper	Trait	Number of individuals	Number of markers extracted—FCR^1^	Number of markers extracted—RFI^2^
Belous et al. (2019)	FCR	715	31	—
Ding et al. (2017)	FCR	338	3	—
Fu et al. (2020)	FCR and RFI	296	15	21
Horodyska et al. (2017)	FCR	952	10	—
Li et al. (2022)	FCR and RFI	485 (FCR) 880 (RFI)	2	22
Miao et al. (2021)	FCR	3,672	60	—
Reyer et al. (2017)	FCR	846	12	—
Sahana et al. (2013)	FCR	3,071	9	—
Wang et al. (2015)	FCR	796	4	—
Bai et al. (2017)	RFI	217	—	12
Ding et al. (2018)	FCR and RFI	1,008	14	14
Do et al. (2014)	RFI	596	—	19
Onteru et al. (2013)	RFI	1,410	—	8
Total number of genomic markers extracted			160	96

^1^Feed conversion ratio.

^2^Residual feed intake.

### Meta-analysis

Considering the 13 papers selected in the systematic review, 160 SNPs for FCR and 96 SNPs for RFI were identified. No SNPs were identified to be associated with FCR or RFI in more than one study and 11 markers were associated with both traits. The updated genomic locations of the SNPs are presented in Table S1.

The 160 SNPs for FCR were located on *Sus scrofa* chromosomes (**SSC**) SSC1, SSC2, SSC3, SSC4, SSC5, SSC6, SSC7, SSC9, SSC11, SSC12, SSC13, SSC14, SSC15, and SSC18. The 96 SNPs for RFI were located on SSC1, SSC2, SSC3, SSC4, SSC5, SSC6, SSC7, SSC8, SSC9, SSC10, SSC11, SSC12, SSC13, SSC14, SSC15, SSC16, and SSC17. SSC5 and SSC2 presented the highest number of SNPs associated with FCR and RFI, respectively. Table S2 shows the number of SNPs associated with the 2 traits studied. The SNPs were considered significant if its *P*-value was less than 3.125 × 10^-4^ and 5.208 × 10^-4^ for FCR and RFI, respectively.

Out of the 160 SNPs extracted from the studies related to FCR, 145 were considered significantly associated with FCR based on the meta-analysis. These SNPs represent a strong association with FCR in pigs, and their respective *P*-values were below the established significance thresholds. The 5 SNPs with the lowest *P*-values, indicating the strongest associations, were: rs80896554 (SSC 15; *P*-value: 3.981 × 10^−8^), rs80955217 (SSC14; *P*-value: 9.036 × 10^-9^), rs80807306 (SSC14; *P*-value: 2.138 × 10^-8^), rs80938302 (SSC14; *P*-value: 9.268 × 10^-8^), and rs80840893 (SSC14; *P*-value: 2.037 × 10^-7^). A detailed list of all significant SNPs and their respective *P*-values is provided in Table S3.

In contrast, the 15 SNPs reported by previous studies that did not reach the significance level of the meta-analysis (potential false positives) are: rs334464676 (SSC17), rs336083023 (SSC4), rs340527699 (SSC4), rs345794390 (SSC13), rs698819984 (SSC17), rs80812508 (SSC14), rs80844227 (SSC14), rs80883237 (SSC7), rs80895086 (SSC7), rs80904397 (SSC14), rs80934608 (SSC15), rs81245790 (SSC18), rs81332374 (SSC9), rs81454344 (SSC15), and rs81467760 (SSC18).

Regarding the RFI trait, 90 out of the 96 SNPs extracted were significantly associated. The 5 SNPs with the lowest *P*-values, indicating the strongest associations, were rs80782607 (SSC1; *P*-value: 3.8 × 10^-8^), rs80810051 (SSC15; *P*-value: 4.6 × 10^-7^), rs80836254 (SSC5; *P*-value: 9.94 × 10^-7^), rs81270180 (SSC13; *P*-value: 1.47 × 10^-7^), and rs81314967 (SSC10; *P*-value: 2.43 × 10^-7^). The potential false positive SNPs are rs333944426 (SSC9), rs80875559 (SSC5), rs81258024 (SSC9), rs81311244 (SSC8), rs81402975 (SSC8), and rs81430022 (SSC11). The list of all SNPs considered significant, along with their respective *P*-values, is shown in Table S3.

### Gene annotation, functional analysis, and gene networks

For FCR, 22 out of the 145 significant SNPs overlapped with more than one gene, and 33 SNPs were not harbored by any gene. The total number of genes identified for FCR was 145. For RFI, 34 SNPs overlapped with more than one gene, and 25 SNPs were not associated with any gene. The total number of genes identified for RFI was 133. Only 3 genes were identified for both traits: ENSSSCG00000054980, ENSSSCG00000056451, and ENSSSCG00000061170. The complete list of genes is shown in Table S4.

The functional enrichment analyses performed on the genes identified for FCR revealed 16 GO terms that were considered statistically significant (*P* < 0.05), including 9 biological processes, 4 cellular components, and 3 molecular functions. The enrichment analysis identified 19 genes that contributed to the significance of these 16 GO terms. For RFI, 6 statistically significant GO terms were identified (*P* < 0.05), including 3 biological processes, one cellular component, and 2 molecular functions. Of the 113 candidate genes, 15 contributed to the significance of these GO terms. Tables 2 and 3 show the significant terms identified, their respective *P*-values, and associated genes for FCR and RFI, respectively. Information on all the GO terms can be found in Table S5. The key GO terms are: actin cytoskeleton (GO_BP:0030036), membrane (GO_CC:0016020), integral components of the peroxisomal membrane (GO_CC:0005779), and carbohydrate-binding (GO_MF:0030246).

**Table 2.** Significant results of enrichment analyses related to the FCR

Category	Term	Count	*P*-value	Genes
GO: biological process	GO:0061386 closure of optic fissure	2	0.009	*MED18*, *PHACTR4*
GO: biological process	GO:2001045 negative regulation of integrin-mediated signaling pathway	2	0.012	*MED18*, *PHACTR4*
GO: biological process	GO:0033598 mammary gland epithelial cell proliferation	2	0.021	*TNFSF11*, *BTRC*
GO: biological process	GO:0043085 positive regulation of catalytic activity	2	0.045	*MED18*, *PHACTR4*
GO: biological process	GO:0048484 enteric nervous system development	2	0.045	*MED18*, *PHACTR4*
GO: biological process	GO:0001843 neural tube closure	3	0.010	*MED18*, *SUFU*, *PHACTR4*
GO: biological process	GO:0030036 actin cytoskeleton organization	4	0.006	*DLC1*, *WASF2*, *MED18*, *PHACTR4*
GO: biological process	GO:0051726 regulation of cell cycle	4	0.009	*MED18*, *YEATS4*, *PHACTR4*, *BTRC*
GO: biological process	GO:0007165 signal transduction	5	0.044	*DLC1*, *TRHDE*, *ARHGAP27*, *TCP11L1*, *TENM3*
GO: cellular component	GO:0000178 exosome (RNase complex)	2	0.034	*DIS3L2*, *ZFC3H1*
GO: cellular component	GO:0005779 integral component of peroxisomal membrane	2	0.048	*FIS1*, *FAR2*
GO: cellular component	GO:0030027 lamellipodium	3	0.045	*WASF2*, *MED18*, *PHACTR4*
GO: cellular component	GO:0016020 membrane	8	0.012	ENSSSCG00000056202, *DLC1*, ENSSSCG00000034739, *TRHDE*, *FRS2*, *ABCC2*, *TNFSF4*, ENSSSCG00000008665
GO: molecular function	GO:0072542 protein phosphatase activator activity	2	0.023	*MED18*, *PHACTR4*
GO: molecular function	GO:0003779 actin-binding	4	0.034	ENSSSCG00000056202, *WASF2*, *MED18*, *PHACTR4*
Molecular function	KW-0009 actin-binding	4	0.007	ENSSSCG00000056202, *WASF2*, *MED18*, *PHACTR4*

**Table 3.** Significant results of enrichment analyses related to the RFI in pigs

Category	Term	Count	*P*-value	Genes
GO: biological process	GO:0000724 double-strand break repair via homologous recombination	3	0.020	*SAMHD1*, *RAD51B*, *ZFYVE26*
GO: biological process	GO:0030520 intracellular estrogen receptor signaling pathway	2	0.037	*SAFB*, *POU4F2*
GO: biological process	GO:0050684 regulation of mRNA processing	2	0.041	*SAFB*, *SAFB2*
GO: cellular component	GO:0005736 DNA-directed RNA polymerase I complex	2	0.035	*POLR1D*, *LOC106505238*
GO: molecular function	GO:0030246 carbohydrate-binding	5	0.001	*ASGR1*, *ASGR2*, *MAN2B1*, ENSSSCG00000040377, *ADGRL2*
GO: molecular function	GO:0003677 DNA-binding	9	0.031	*HIPK1*, ENSSSCG00000013715, *ILF3*, *RAD51B*, *NFIX*, *POLR1D*, *PHTF1*, *RFX2*, *LOC106505238*

Gene interaction networks were constructed using GeneMANIA (Warde-Farley et al., 2010) as a plug-in for Cytoscape (Shannon et al., 2003). These analyses aimed to investigate the interactions between genes related to FCR and RFI, since this knowledge is fundamental to the understanding of the biological underlying mechanisms.

**Figure 2.**
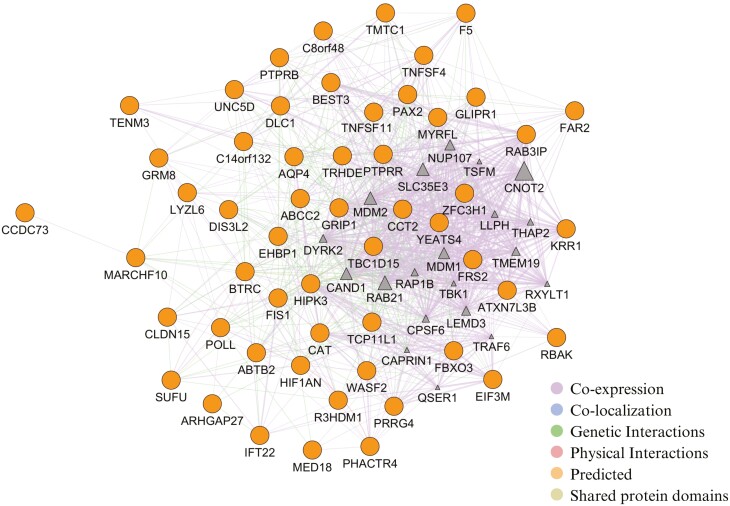
Gene network analysis for FCR. The diagram shows the genetic interaction network associated with FCR in pigs. Ellipse nodes represent genes associated with FCR, while triangle nodes indicate functionally similar genes identified by the software in previously published papers. The edges represent interactions or relationships between the genes.

Fifty-four genes were used for the gene network analyses associated with FCR. The network was made up of 74 nodes representing the individual genes since 20 other functionally similar genes (triangle) were identified by the software since GeneMANIA presents genes that have properties shared with the genes in the initial query. The network also showed 1,066 edges, which represent the interactions or relationships between the genes. Of these edges, the majority were co-expression and genetic interaction, as shown in Figure 2. All the genes included in the network interacted with each other.

**Figure 3.**
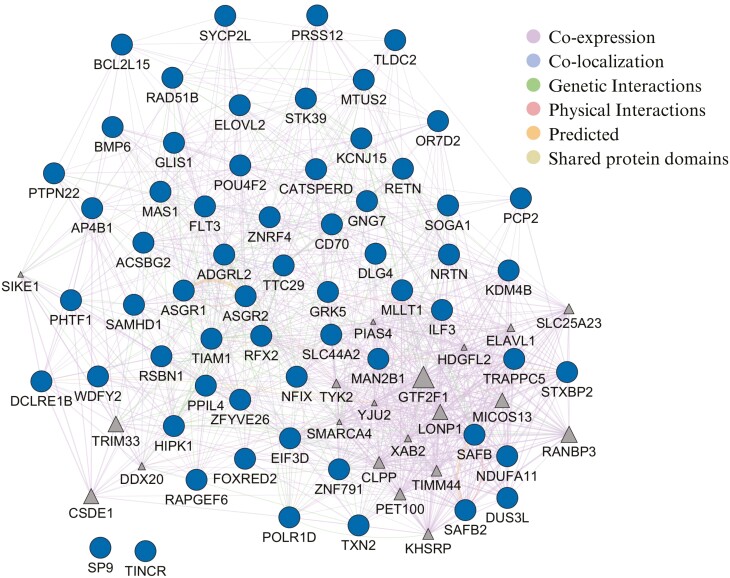
Gene network analysis for RFI. The diagram shows the genetic interaction network associated with RFI in pigs. Ellipse nodes represent genes associated with RFI, while triangle nodes indicate functionally similar genes identified by the software in previously published papers. The edges represent interactions or relationships between the genes.

The gene network associated with RFI was built with 82 nodes: 62 genes associated with significant SNPs after meta-analysis and 20 other functionally similar genes (triangle). This network showed 962 edges and most of them were co-expression and genetic interactions (Figure 3). In this gene network, most of the genes showed some kind of interaction with another gene, but the gene TINCR ubiquitin domain containing (*TINCR*), and Sp9 Transcription Factor (*SP9*) genes were not linked to any other gene.

**Figure 4.**
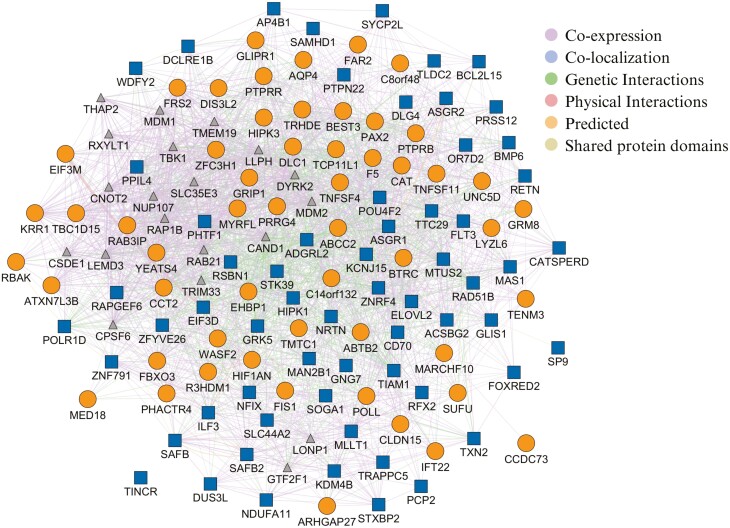
Gene network analysis for both FCR and RFI traits. The diagram shows the genetic interaction network associated with FCR and RFI in pigs. Ellipse nodes represent genes associated with FCR, while square nodes represent genes associated with RFI. Triangle nodes indicate functionally similar genes identified by the software in previously published papers. The edges represent interactions or relationships between the genes.

The third network constructed combined the 54 and 62 genes associated with FCR and RFI, respectively, as well as 20 other functionally similar genes, resulting in 136 nodes in the network and 2,531 edges, the majority of which are co-expression and genetic interaction (Figure 4). Most of the genes identified for RFI and FCR were connected in the gene network, but the *TINCR* gene, associated with RFI, was not connected with any other gene.

## Discussion

The systematic review carefully followed the eligibility criteria for papers. Only studies that contained all the necessary information were selected for inclusion in our database. Therefore, this selection process resulted in a significant reduction in the number of papers selected from the search platforms throughout the filtering process. A genomic meta-analysis and an enrichment analysis for FCR and RFI were carried out according to the results of 10 studies for FCR and 6 studies for RFI. We evaluated the existing knowledge about the genomic regions associated with the studied traits, with the main objective of identifying and validating genes associated with them.

After extracting the significant SNPs from each study, 160 SNPs for FCR were identified. A descriptive analysis of these data revealed that there were no significant SNPs in common between different reports, showing a possible inconsistency across studies. For RFI, 96 significant SNPs were extracted from the papers, and similar to FCR, there were no significant SNPs in common between studies. The absence of overlapping SNPs between studies highlights the complexity of the genetic architecture underlying FCR and RFI in pigs, which may differ across populations, experimental designs, and statistical approaches. A meta-analysis enables the integration of results from diverse studies, offering a broader perspective on potential genomic regions of interest. Furthermore, it facilitates the identification of candidate genes associated with these traits, even when specific SNPs may vary. This approach enhances the robustness of the conclusions drawn from individual studies. The ability to detect significant associations in GWAS depends on various factors such as sample size (Fu et al., 2020; Uffelmann et al., 2021). The sample sizes used in the studies included in this meta-analysis ranged from 217 to 3,672 individuals. The relatively small sample sizes from some of the studies (Table 1) may have contributed to the lack of common SNPs or genes across studies and traits. In addition to sample size, other factors that may explain the lack of overlap among the studies include the type and density of the SNP panels used to genotype the animals, the studied populations that may differ, the quality control of the data, GWAS methods used, multiple testing correction methods, and the significance threshold levels.

When comparing the significant markers extracted for both FCR and RFI, only 11 SNPs were present in the 2 databases. However, these 11 SNPs were extracted from the same study (i.e., Ding et al., 2018), which performed a GWAS for both traits. Although there are several GWAS for FCR and RFI in pigs, they have relatively small sample sizes. This disparity in sample sizes across studies highlights the need for caution when interpreting the results, since studies with small sample sizes may be more susceptible to false negatives. Therefore, GWAS with larger sample sizes for FCR and RFI in pigs are still needed.

The meta-analysis identified 145 significant SNPs for FCR and 90 for RFI in different regions of the pig genome. Of these, the 11 markers present in both databases were significant for both traits, all located on SSC1 and belonging to the study of Ding et al. (2018), as previously described. However, due to the high genetic correlation between FCR and RFI, observed in studies such as that of Do et al. (2013), with values ranging from 0.85 to 0.91, and that of Saintilan et al. (2011), which reported a correlation of 0.85, we expected a greater overlap in the SNPs identified for both traits. This lack of overlap may suggest great complexity in the genetic mechanisms underlying these traits.

The process of annotating genes nearby (32 Kb window) the significant SNPs in the meta-analysis resulted in a total of 105 non-duplicated genes for FCR. For RFI, 114 non-duplicated genes were identified. It is important to note that 33 and 25 significant markers after the meta-analysis did not have any genes associated with FCR and RFI, respectively. Another notable aspect to highlight is that the duplicated genes for each trait were associated with SNPs taken from the same study. In other words, we did not find any genes associated with markers from different studies. In addition, of those 105 and 114 genes annotated for FCR and RFI, respectively, only 62 and 54 had complete identification and symbol information, which may be attributed to the lack of complete information on the pig genome, highlighting the need for further research to elucidate these genes in a more comprehensive manner.

The annotated gene sets were used to perform enrichment analyses and to build the gene networks. The discussion focused primarily on 4 GO terms that were more directly related to the biological processes underlying the traits.

For FCR, the genes Mediator Complex Subunit 18 (*MED18*) and Phosphatase and Actin Regulator 4 (*PHACTR4*) were associated with 11 GO terms, including the positive regulation of catalytic activity (GO_BP:0043085), the molecular function of actin-binding (KW-0009 and GO_MF:0003779), the regulation of the cell cycle (GO_BP:0051726), closure of optic fissure (GO_BP:0061386), neural tube closure (GO_BP:0001843), negative regulation of integrin-mediated signaling pathway (GO_BP:2001045), protein phosphatase activator activity (GO_MF:0072542), to the cellular component lamellipodium (GO_CC:0030027) and enteric nervous system development (GO_BP:0048484). In, these genes showed a co-expression interaction, i.e., the expression levels were similar between different gene expression studies.

The *MED18* gene, a component of the mediator complex, has been previously reported in the literature to regulate transcription and is considered a candidate gene influencing FCR in pigs (Belous et al., 2019). Mediator modules are required for the regulation of different subsets of genes, in which *MED18* is associated with the formation of a stable initiation complex, efficient basal transcription, and activated transcription (Larivière et al., 2008). This may explain its association with several GO terms since it can influence the expression of other genes.

*PHACTR4* has also been reported in the literature as a candidate gene for FCR in pigs (Belous et al., 2019). Its functions include participation in the proliferation of nerve cells, as well as interaction with muscle actin, being involved in the motility of different types of cells. This gene is present in the biological process related to the organization of the actin cytoskeleton (GO_BP:0030036). It is known that the actin cytoskeleton plays a fundamental role in cell movement, which is essential for several physiological processes, including cell growth and proliferation (Svitkina, 2018). Changes in the organization of the actin cytoskeleton may have an impact on these biological processes.

The ATP binding cassette subfamily C member 2 (*ABCC2*) gene associated with the membrane (GO_CC:0016020) is expressed in the main physiological barriers, such as in intestinal cells (Jemnitz et al., 2010). The gastrointestinal epithelium functions as a selective barrier to absorb nutrients, electrolytes, and water (Groschwitz and Hogan, 2009). *ABCC2* encodes a membrane transporter involved in the transportation of various substances and is important for the intestinal barrier as well as its regulation (Arana et al., 2016). Thus, alterations in its expression or activity may affect the absorption of nutrients or the excretion of diet-related metabolites, influencing the animals’ feed efficiency.

Other genes also associated with the membrane (GO_CC:0016020) were Thyrotropin-Releasing Hormone Degrading Enzyme (*TRHDE*) and Fibroblast Growth Factor Receptor Substrate 2 (*FRS2*), which have been previously associated with FCR in pigs (Miao et al., 2021). *TRHDE* is an extracellular peptidase that specifically degrades thyrotropin-releasing hormone (**TRH**) to regulate appetite and metabolism (Schomburg et al., 1999; Freudenberg et al., 2011). *FRS2* is involved in fibroblast growth factor 21 (FGF21) signaling with AMP-activated protein kinase (AMPK) and is related to energy metabolism (Videla et al., 2018). *FGF21* regulates whole-body energy metabolism (Salminen et al., 2017) and AMPK is an important metabolic energy sensor in cells, regulating energy production by stimulating the breakdown of glucose and lipids (Lage et al., 2008; Hardie et al., 2012). Therefore, *TRHDE* and *FRS2* are promising candidate genes for FCR, given their participation in important processes related to feed efficiency in pigs.

Fatty Acyl-CoA Reductase 2 (*FAR2*) is a key gene associated with β-oxidation of fatty acids and translocation of acetyl-CoA (Miao et al., 2021), and it has been associated with insulin resistance in humans (Burghardt et al., 2016). This gene is associated with integral components of the peroxisomal membrane (GO_CC:0005779). Peroxisomes are specialized cellular structures involved in various metabolic functions, including lipid metabolism (Périchon et al., 1998). Lipid metabolism can explain variation in FCR (Xu et al., 2018), so *FAR2* could be a candidate gene for the trait.

The Fission, Mitochondrial 1 (*FIS1*) gene, also associated with integral components of the peroxisomal membrane (GO_CC:0005779), is known to play a role in mitochondrial dynamics (Onterou et al., 2013). Kobayashi et al. (2007) have shown a possible relationship of *FIS1* with peroxisomes. Thus, this gene may be related to the morphology and division of peroxisomes in mammalian cells.

The 3 genes that were associated with the membrane (*ABCC2*, *TRHDE*, and *FRS2*) and the 2 genes associated with integral components of the peroxisomal membrane (*FAR2* and *FIS1*) showed interaction in the gene network for FCR. *ABCC2* had a co-expression interaction with *FRS2*, *FAR2*, and *FIS1*. *TRHDE* also showed a co-expression interaction with *FRS2* and *FAR2*. This co-expression between these genes indicates that 2 genes are linked when their expression levels are similar in different conditions of a gene expression study, showing a possible functional interaction between them and their participation in similar processes.

In this study, we identified candidate genes that may be associated with FCR in pigs. These genes were selected based on their function in biological processes related to the studied trait. Notably, genes such as *MED18*, *PHACTR4*, *ABCC2*, *TRHDE*, *FRS2*, *FAR2*, and *FIS1* were identified as possible contributors to FCR variation, with strong biological relevance, with the *ABCC2* gene having not yet been previously associated with this trait. These findings contribute to a more comprehensive understanding of the genetic mechanisms underlying FCR, suggesting new targets for future research.

Carbohydrates are biomolecules involved in many vital processes. To deal with this multiplicity of functions, several carbohydrate-active proteins have acquired non-catalytic modules that interact specifically with mono, oligo, and polysaccharides. These modules allow these proteins to recognize and bind specifically to carbohydrates, facilitating their various biological functions. In addition, they can be found, for example, in proteins that recognize polysaccharides such as cellulose, chitin, β-glucans, starch, glycogen, and in active enzymes (Hashimoto, 2006; Guillén et al., 2010; Armenta et al., 2017).

In the context of RFI, this carbohydrate-binding capacity may be related to the interaction between proteins or enzymes and the carbohydrates present in food, as it may be an important event for processes related to the metabolism of these important components of the animal diet (Guillén et al., 2010). For example, in the study of Bueren and Boraston (2007), the authors pointed out that the recognition of starch by these binding modules is important for the activity of starch-degrading enzymes, which shows an important molecular function associated with RFI.

Genes such as Adhesion G Protein-Coupled Receptor (*ADGRL2)*, Asialoglycoprotein Receptor 1 (*ASGR1)*, Asialoglycoprotein Receptor 2 (*ASGR2)*, and Mannosidase Alpha Class 2B Member 1 (*MAN2B1)* were associated with this molecular function of carbohydrate-binding, making them important and promising candidate genes for RFI. They also showed interactions in the gene networks: *ADGRL2* and *MAN2B1* had co-expression interactions with *ASGR1* and *ASGR2*, which means that, in gene expression studies, their expression levels were similar; and *AGR1* interacted with *AGR2* through co-expression and physical interaction.

*ADGRL2* encodes a protein that belongs to the family of G protein-coupled receptors (Hamann et al., 2015). *ASGR1* is a transmembrane protein that plays a critical role in serum glycoprotein homeostasis (Bai et al., 2017). *ASGR1* encodes asialoglycoprotein receptor 1 and is highly expressed in the liver; *ASGR2* encodes receptor 2, and both genes mediate the removal of this glycoconjugate, which is potentially hazardous to health (Soukharev et al., 2000; Harris et al., 2012). The *MAN2B1* gene encodes the α-D-mannosidase enzyme, which acts in the degradation of N-linked oligosaccharides and therefore plays an important role in the mannose metabolism in mammals (Khan and Ranganathan, 2009).

The genes *ADGRL2*, *ASGR1*, *ASGR2*, and *MAN2B1* are important candidate genes for RFI. These results provide new insights into the understanding of the genetic mechanisms regulating feed efficiency in pigs. These findings contribute significantly to the understanding of the genetic background of feed efficiency in pigs, expanding the pool of candidate genes and paving the way for future research that could explore their potential in pig breeding programs.

The biological process of repairing DNA double-strand breaks through homologous recombination (GO_BP:0000724) is an important way of repairing DNA damage caused by double-strand breaks, which are considered a major disruption to genetic integrity (Arnaudeau et al., 2001). Efficient DNA repair is essential to guarantee the integrity and stability of the genome. Another important factor for genome integrity is the molecular function of DNA-binding proteins (GO_MF:0003677), which play essential roles in biological processes such as gene expression and DNA repair (Corona and Guo, 2016). In this context of gene expression, the biological process of regulation of messenger RNA processing (GO_BP:0050684) is also important. The genes that have been associated with these biological processes and molecular functions are: RAD51 Paralog B (*RAD51B),* SAM and HD Domain Containing Deoxynucleoside Triphosphate Triphosphohydrolase 1 (*SAMHD1*), Zinc Finger FYVE-type Containing 26 (*ZFYVE26*), Homeodomain Interacting Protein Kinase *1* (*HIPK1*), Interleukin Enhancer Binding Factor 3 (*ILF3*), Nuclear Factor I X (*NFIX*), Putative Homeodomain Transcription Factor 1 (*PHTF1*), RNA Polymerase I and III subunit D (*POLR1D*), Regulatory factor X2 (*RFX2*), Scaffold Attachment Factor B (*SAFB*), and Scaffold Attachment Factor B2 (*SAFB2*). Although they have no known direct link to RFI, they may be influencing the expression of genes associated with the trait.

Interestingly, there was no overlap between the FCR and RFI traits in terms of enrichment analysis and only 3 genes were identified for both traits, but none of those genes were associated with processes or functions in the enrichment analysis. However, due to the high genetic correlation between FCR and RFI in pigs (0.85 to 0.91 Saintilan et al. 2011; Do et al., 2013; 0.85), a greater sharing of markers and genes between them was expected. This lack of overlap may indicate the complexity of the feed efficiency measures studied, which makes it challenging to identify the same genes or processes associated with both traits, despite the known relationship between them. The genetic background of the traits may be affected by different genomic regions, reflecting the genetic diversity underlying them. In addition, it is important to consider the possible limitations of the analyses, mainly due to the inconsistencies found in the results extracted from each GWAS.

These limitations became evident while conducting this GWAS meta-analysis, such as the small sample sizes used in individual studies, the lack of complete information in many articles, and the restricted access to the full datasets of markers used in the analyses. Such data would have the potential to enrich and expand our findings. Additionally, the absence of overlapping genomic regions across studies for the same trait further highlights the challenges in achieving consensus in this field, which is not surprising for highly polygenic traits. Despite these limitations, this meta-analysis provides a foundation for further exploration of the genes identified as potential candidates, offering valuable perspectives for future research.

## Conclusions

Our meta-analysis of the results of GWAS helped identify possible false positive markers that had been reported in individual GWAS, considering that of the 160 and 96 significant SNPs extracted for FCR and RFI, respectively, 15 and 6 markers were not significant after meta-analysis. The most relevant candidate genes identified for FCR are *MED18*, *PHACTR4*, *ABCC2*, *TRHDE*, *FRS2*, *FAR2*, and *FIS1*, whereas for RFI, the genes *ADGRL2*, *ASGR1*, *ASGR2*, and *MAN2B1* were selected as candidate genes. The biological processes identified also contributed to a better understanding of the genomic background of feed efficiency traits in pigs.

## Supplementary Material

skaf010_suppl_Supplementary_Material

## Abbreviations

AMPK: AMP-activated protein kinase
DAVID: Database for Annotation, Visualization, and Integrated Discovery
FCR: feed conversion ratio
FGF21: fibroblast growth factor 21
GO: gene ontology
GWAS: genome-wide association studies
QTL: quantitative trait loci
RFI: residual feed intake
SNP: single nucleotide polymorphism
TRH: thyrotropin-releasing hormone

## References

[CIT0001] Arana, M. R., G. N. Tocchetti, J. P. Rigalli, A. D. Mottino, and S. S. Villanueva. 2016. Physiological and pathophysiological factors affecting the expression and activity of the drug transporter MRP2 in intestine. Impact on its function as membrane barrier. Pharmacol. Res. 109:32–44. doi: https://doi.org/10.1016/j.phrs.2016.04.014 27109321

[CIT0002] Armenta, S., S. Moreno-Mendieta, Z. Sánchez-Cuapio, S. Sánchez, and R. Rodríguez-Sanoja. 2017. Advances in molecular engineering of carbohydrate-binding modules. Proteins. 85:1602–1617. doi: https://doi.org/10.1002/prot.25327 28547780

[CIT0003] Arnaudeau, C., C. Lundin, and T. Helleday. 2001. DNA double-strand breaks associated with replication forks are predominantly repaired by homologous recombination involving an exchange mechanism in mammalian cells. J. Mol. Biol. 307:1235–1245. doi: https://doi.org/10.1006/jmbi.2001.4564 11292338

[CIT0004] Bai, C., Y. Pan, D. Wang, F. Cai, S. Yan, Z. Zhao, and B. Sun. 2017. Genome-wide association analysis of residual feed intake in Junmu No. 1 White pigs. Anim. Genet. 48:686–690. doi: https://doi.org/10.1111/age.12609 29076177

[CIT0005] Belous, A. A., A. A. Sermyagin, O. V. kostyunina, G. Brem, and N. A. Zinovieva. 2019. Study of genetic architecture of feed conversion rate in duroc young boars (sus scrofa) based on the genome-wide SNP analysis. Agric. Biol. 54:705–712. doi: https://doi.org/10.15389/agrobiology.2019.4.705rus

[CIT0006] Brito, L. F., H. R. Oliveira, K. Houlahan, P. A. Fonseca, S. Lam, A. M. Butty, D. J. Seymour, G. Vargas, T. C. Chud, F. F. Silva, et al 2020. Genetic mechanisms underlying feed utilization and implementation of genomic selection for improved feed efficiency in dairy cattle. Can. J. Anim. Sci. 100:587–604. doi: https://doi.org/10.1139/cjas-2019-0193

[CIT0007] Bueren, A. L. V., and A. B. Boraston. 2007. The structural basis of α-glucan recognition by a family 41 carbohydrate-binding module from thermotoga maritima. J. Mol. Biol. 365:555–560. doi: https://doi.org/10.1016/j.jmb.2006.10.018 17095014

[CIT0008] Burghardt, K. J., J. M. Goodrich, D. C. Dolinoy, and V. L. Ellingrod. 2016. Gene-specific DNA methylation may mediate atypical antipsychotic-induced insulin resistance. Bipolar Disord. 18:423–432. doi: https://doi.org/10.1111/bdi.12422 27542345 PMC5322870

[CIT0009] Corona, R. I., and J. T. Guo. 2016. Statistical analysis of structural determinants for protein-DNA-binding specificity. Proteins. 84:1147–1161. doi: https://doi.org/10.1002/prot.25061 27147539 PMC4945413

[CIT0010] De Bakker, P. I., M. A. Ferreira, X. Jia, B. M. Neale, S. Raychaudhuri, and B. F. Voight. 2008. Practical aspects of imputation-driven meta-analysis of genome-wide association studies. Hum. Mol. Genet. 17:R122–R128. doi: https://doi.org/10.1093/hmg/ddn288 18852200 PMC2782358

[CIT0011] Ding, R., J. Quan, M. Y. X. Wang, E. Zheng, H. Yang, D. Fu, Y. Yang, L. Yang, Z. Li, D. Liu, et al 2017. Genome-wide association analysis reveals genetic loci and candidate genes for feeding behavior and eating efficiency in Duroc boars. PLoS One. 12:e0183244. doi: https://doi.org/10.1371/journal.pone.0183244 28813538 PMC5559094

[CIT0012] Ding, R., M. Yang, X. Wang, J. Quan, Z. Zhuang, S. Zhou, S. Li, Z. Xu, E. Zheng, G. Cai, et al 2018. Genetic architecture of feeding behavior and feed efficiency in a Duroc pig population. Front. Genet. 9:220. doi: https://doi.org/10.3389/fgene.2018.00220 29971093 PMC6018414

[CIT0013] Do, D. N., A. B. Strathe, J. Jensen, T. Mark, and H. N. Kadarmideen. 2013. Genetic parameters for different measures of feed efficiency and related traits in boars of three pig breeds. J. Anim. Sci. 91:4069–4079. doi: https://doi.org/10.2527/jas.2012-6197 23825329

[CIT0061] Do, D. N., A. B. Strathe, T. Ostersen, S. D. Pant, and H. N. Kadarmideen. 2014. Genome-wide association and pathway analysis of feed efficiency in pigs reveal candidate genes and pathways for residual feed intake. Front. Genet. 5:307. doi: https://doi.org/10.3389/fgene.2014.00307 25250046 PMC4159030

[CIT0014] Duarte, D. A. S., C. J. Newbold, E. Detmann, F. F. Silva, P. H. F. Freitas, R. Veroneze, and M. S. Duarte. 2019. Genome-wide association studies pathway-based meta-analysis for residual feed intake in beef cattle. Anim. Genet. 50:150–153. doi: https://doi.org/10.1111/age.12761 30644110

[CIT0015] Durinck, S., Y. Moreau, A. Kasprzyk, S. Davis, B. de Moor, A. Brazma, and W. Huber. 2005. BioMart and bioconductor: a powerful link between biological databases and microarray data analysis. Bioinformatics. 21:3439–3440. doi: https://doi.org/10.1093/bioinformatics/bti525 16082012

[CIT0016] Evangelou, E., and J. P. A. Ioannidis. 2013. Meta-analysis methods for genome-wide association studies and beyond. Nat. Rev. Genet. 14:379–389. doi: https://doi.org/10.1038/nrg3472 23657481

[CIT0017] Freudenberg, J., H. S. Lee, B. G. Han, H. D. Shin, Y. M. Kang, Y. K. Sung, S. C. Shim, C. B. Choi, A. T. Lee, P. K. Gregersen, et al 2011. Genome-wide association study of rheumatoid arthritis in Koreans: population-specific loci as well as overlap with European susceptibility loci. Arthritis. Rheum. 63:884–893. doi: https://doi.org/10.1002/art.30235 21452313

[CIT0018] Fu, L., Y. Jiang, C. Wang, M. Mei, Z. Zhou, Y. Jiang, H. Song, and X. Ding. 2020. A genome-wide association study on feed efficiency related traits in Landrace pigs. Front. Genet. 11:692. doi: https://doi.org/10.3389/fgene.2020.00692 32719719 PMC7350416

[CIT0019] Groschwitz, K. R., and S. P. Hogan. 2009. Intestinal barrier function: molecular regulation and disease pathogenesis. J. Allergy Clin. Immunol. 124:3–20; quiz 21. doi: https://doi.org/10.1016/j.jaci.2009.05.038 19560575 PMC4266989

[CIT0020] Guillén, D., S. Sánchez, and R. Rodríguez-Sanoja. 2010. Carbohydrate-binding domains: multiplicity of biological roles. Appl. Microbiol. Biotechnol. 85:1241–1249. doi: https://doi.org/10.1007/s00253-009-2331-y 19908036

[CIT0021] Hamann, J., G. Aust, D. Araç, F. B. Engel, C. Formstone, R. Fredriksson, R. A. Hall, B. L. Harty, C. Kirchhoff, B. Knapp, et al 2015. International union of basic and clinical pharmacology. XCIV. Adhesion G protein-coupled receptors. Pharmacol. Rev. 67:338–367. doi: https://doi.org/10.1124/pr.114.009647 25713288 PMC4394687

[CIT0022] Hardie, D. G., F. A. Ross, and S. A. Hawley. 2012. AMPK: a nutrient and energy sensor that maintains energy homeostasis. Nat. Rev. Mol. Cell Biol. 13:251–262. doi: https://doi.org/10.1038/nrm3311 22436748 PMC5726489

[CIT0023] Harris, R. L., C. W. Van den Berg, and D. J. Bowen. 2012. ASGR1 and ASGR2, the genes that encode the asialoglycoprotein receptor (ashwell receptor), are expressed in peripheral blood monocytes and show interindividual differences in transcript profile. Mol. Biol. Int. 2012:283974. doi: https://doi.org/10.1155/2012/283974 22919488 PMC3419429

[CIT0024] Hashimoto, H. 2006. Recent structural studies of carbohydrate-binding modules. Cell. Mol. Life Sci. 63:2954–2967. doi: https://doi.org/10.1007/s00018-006-6195-3 17131061 PMC11136102

[CIT0025] Horodyska, J., R. M. Hamill, P. F. Varley, H. Reyer, and K. Wimmers. 2017. Genome-wide association analysis and functional annotation of positional candidate genes for feed conversion efficiency and growth rate in pigs. PLoS One. 12:e0173482. doi: https://doi.org/10.1371/journal.pone.0173482 28604785 PMC5467825

[CIT0026] Huang, W., B. T. Sherman, and R. A. Lempicki. 2009. Systematic and integrative analysis of large gene lists using DAVID bioinformatics resources. Nat. Protoc. 4:44–57. doi: https://doi.org/10.1038/nprot.2008.211 19131956

[CIT0027] Jemnitz, K., K. Heredi-Szabo, J. Janossy, E. Ioja, L. Vereczkey, and P. Krajcsi. 2010. ABCC2/Abcc2: a multispecific transporter with dominant excretory functions. Drug Metab. Rev. 42:402–436. doi: https://doi.org/10.3109/03602530903491741 20082599

[CIT0028] Khan, J. M., and S. Ranganathan. 2009. A multi-species comparative structural bioinformatics analysis of inherited mutations in α-D-Mannosidase reveals strong genotype-phenotype correlation. BMC Genomics. 10:S33. doi: https://doi.org/10.1186/1471-2164-10-S3-S33 PMC2788387 19958498

[CIT0029] Kobayashi, S., A. Tanaka, and Y. Fujiki. 2007. Fis1, DLP1, and Pex11p coordinately regulate peroxisome morphogenesis. Exp. Cell Res. 313:1675–1686. doi: https://doi.org/10.1016/j.yexcr.2007.02.028 17408615

[CIT0030] Korte, A., and A. Farlow. 2013. The advantages and limitations of trait analysis with GWAS: a review. Plant Methods. 9:29. doi: https://doi.org/10.1186/1746-4811-9-29 23876160 PMC3750305

[CIT0031] Koutsos, T. M., G. C. Menexes, and C. A. Dordas. 2019. An efficient framework for conducting systematic literature reviews in agricultural sciences. Sci. Total Environ. 682:106–117. doi: https://doi.org/10.1016/j.scitotenv.2019.04.354 31108265

[CIT0032] Kronenberg, F. 2008. Genome-wide association studies in aging-related processes such as diabetes mellitus, atherosclerosis and cancer. Exp. Gerontol. 43:39–43. doi: https://doi.org/10.1016/j.exger.2007.09.005 17967522

[CIT0033] Lage, R., C. Diéguez, A. Vidal-Puig, and M. López. 2008. AMPK: a metabolic gauge regulating whole-body energy homeostasis. Trends Mol. Med. 14:539–549. doi: https://doi.org/10.1016/j.molmed.2008.09.007 18977694

[CIT0034] Larivière, L., M. Seizl, S. van Wageningen, S. Röther, L. van de Pasch, H. Feldmann, K. Strässer, S. Hahn, F. C. Holstege, and P. Cramer. 2008. Structure-system correlation identifies a gene regulatory mediator submodule. Genes Dev. 22:872–877. doi: https://doi.org/10.1101/gad.465108 18381891 PMC2279198

[CIT0035] Li, W., Z. Wang, S. Luo, J. Wu, L. Zhou, and J. Liu. 2022. Genome-wide association analysis and genetic parameters for feed efficiency and related traits in Yorkshire and Duroc pigs. Animals (Basel). 12:1902. doi: https://doi.org/10.3390/ani12151902 35892552 PMC9329986

[CIT0036] Magalhães, A. F. B., G. M. F. Camargo, G. A. F. Junior, D. G. M. Gordo, R. L. Tonussi, R. B. Costa, R. Espigolan, R. M. O. Silva, T. Bresolin, W. B. F. Andrade, et al 2016. Genome-wide association study of meat quality traits in Nellore cattle. PLoS One. 11:e0157845. doi: https://doi.org/10.1371/journal.pone.0157845 27359122 PMC4928802

[CIT0037] Miao, Y., Q. Mei, C. Fu, M. Liao, Y. Liu, X. Xu, X. Li, S. Zhao, and T. Xiang. 2021. Genome-wide association and transcriptome studies identify candidate genes and pathways for feed conversion ratio in pigs. BMC Genomics. 22:294. doi: https://doi.org/10.1186/s12864-021-07570-w 33888058 PMC8063444

[CIT0038] Oliveira, L. F., R. Veroneze, K. R. S. Sousa, H. A. Mulim, A. C. Araujo, Y. Huang, J. S. Johnson, and L. F. Brito. 2024. Genomic regions, candidate genes, and pleiotropic variants associated with physiological and anatomical indicators of heat stress response in lactating sows. BMC Genomics. 25:467. doi: https://doi.org/10.1186/s12864-024-10365-4 38741036 PMC11092106

[CIT0039] Onteru, S. K., D. M. Gorbach, J. M. Young, D. J. Garrick, J. C. M. Dekkers, and M. F. Rothschild. 2013. Whole genome association studies of residual feed intake and related traits in the pig. PLoS One. 8:e61756. doi: https://doi.org/10.1371/journal.pone.0061756 23840294 PMC3694077

[CIT0040] Périchon, R., J. M. Bourre, J. F. Kelly, and G. S. Roth. 1998. The role of peroxisomes in aging. Cell. Mol. Life Sci. 54:641–652. doi: https://doi.org/10.1007/s000180050192 9711231 PMC11147293

[CIT0041] Pomar, C., I. Andretta, and A. Remus. 2021. Feeding strategies to reduce nutrient losses and improve the sustainability of growing pigs. Front. Vet. Sci. 8:742220. doi: https://doi.org/10.3389/fvets.2021.742220 34778430 PMC8581561

[CIT0042] Reyer, H., M. Shirali, S. Ponsuksili, E. Murani, P. F. Varley, J. Jensen, and K. Wimmers. 2017. Exploring the genetics of feed efficiency and feeding behaviour traits in a pig line highly selected for performance characteristics. Mol. Genet. Genomics. 292:1001–1011. doi: https://doi.org/10.1007/s00438-017-1325-1 28500374 PMC5594041

[CIT0043] Sahana, G., V. Kadlecová, H. Hornshøj, B. Nielsen, and O. F. Christensen. 2013. A genome-wide association scan in pig identifies novel regions associated with feed efficiency trait. J. Anim. Sci. 91:1041–1050. doi: https://doi.org/10.2527/jas.2012-5643 23296815

[CIT0044] Saintilan, R., I. Mérour, S. Schwob, P. Sellier, J. Bidanel, and H. Gilbert. 2011. Genetic parameters and halothane genotype effect for residual feed intake in Piétrain growing pigs. Livest. Sci. 142:203–209. doi: https://doi.org/10.1016/j.livsci.2011.07.013

[CIT0045] Salminen, A., A. Kauppinen, and K. Kaarniranta. 2017. FGF21 activates AMPK signaling: impact on metabolic regulation and the aging process. J. Mol. Med. (Berl.) 95:123–131. doi: https://doi.org/10.1007/s00109-016-1477-1 27678528

[CIT0046] Sanna, S., A. U. Jackson, R. Nagaraja, C. J. Willer, W. M. Chen, L. L. Bonnycastle, H. Shen, N. Timpson, G. Lettre, G. Usala, et al 2008. Common variants in the GDF5-UQCC region are associated with variation in human height. Nat. Genet. 40:198–203. doi: https://doi.org/10.1038/ng.74 18193045 PMC2914680

[CIT0047] Schomburg, L., S. Turwitt, G. Prescher, D. Lohmann, B. Horsthemke, and K. Bauer. 1999. Human TRH-degrading ectoenzyme cDNA cloning, functional expression, genomic structure and chromosomal assignment. Eur. J. Biochem. 265:415–422. doi: https://doi.org/10.1046/j.1432-1327.1999.00753.x 10491199

[CIT0048] Shannon, P., A. Markiel, O. Ozier, N. S. Baliga, J. T. Wang, D. Ramage, N. Amin, B. Schwikowski, and T. Ideker. 2003. Cytoscape: a software environment for integrated models of biomolecular interaction networks. Genome Res. 13:2498–2504. doi: https://doi.org/10.1101/gr.1239303 14597658 PMC403769

[CIT0049] Sherman, B. T., M. Hao, J. Qiu, X. Jiao, M. W. Baseler, H. C. Lane, T. Imamichi, and W. Chang. 2022. DAVID: a web server for functional enrichment analysis and functional annotation of gene lists (2021 update). Nucleic Acids Res. 50:W216–W221. doi: https://doi.org/10.1093/nar/gkac194 35325185 PMC9252805

[CIT0050] Soukharev, S., W. Berlin, J. A. Hanover, B. Bethke, and B. Sauer. 2000. Organization of the mouse ASGR1 gene encoding the major subunit of the hepatic asialoglycoprotein receptor. Gene. 241:233–240. doi: https://doi.org/10.1016/s0378-1119(99)00493-x 10675034

[CIT0051] Svitkina, T. 2018. The actin cytoskeleton and actin-based motility. Cold Spring Harb. Perspect. Biol. 10:a018267. doi: https://doi.org/10.1101/cshperspect.a018267 29295889 PMC5749151

[CIT0052] Taherkhani, L., M. H. Banabazi, N. EmamJomeh-Kashan, A. Noshary, and I. Imumorin. 2022. The candidate chromosomal regions responsible for milk yield of cow: a GWAS meta-analysis. Animals (Basel). 12:582. doi: https://doi.org/10.3390/ani12050582 35268150 PMC8909671

[CIT0053] Uffelmann, E., Q. Q. Huang, N. S. Munung, J. Vries, Y. Okada, A. R. Martin, H. C. Martin, T. Lappalainen, and D. Posthuma. 2021. Genome-wide association studies. Nat. Rev. Methods Primers. 1:59. doi: https://doi.org/10.1038/s43586-021-00056-9

[CIT0054] Verardo, L. L., M. S. Lopes, S. Wijga, O. Madsen, F. F. Silva, M. A. Groenen, E. F. Knol, P. S. Lopes, and E. G. andS. 2016. After genome-wide association studies: Gene networks elucidating candidate genes divergences for number of teats across two pig populations. J. Anim. Sci. 94:1446–1458. doi: https://doi.org/10.2527/jas.2015-9917 27136004

[CIT0055] Videla, L. A., R. Vargas, B. Riquelme, J. Fernández, and V. Fernández. 2018. Thyroid hormone-induced expression of the hepatic scaffold proteins Sestrin2, β-Klotho, and FRS2α in relation to FGF21-AMPK signaling. Exp. Clin. Endocrinol. Diabetes. 126:182–186. doi: https://doi.org/10.1055/s-0043-115533 28895643

[CIT0056] Wang, K., D. Liu, J. Hernandez-Sanchez, J. Chen, C. Liu, Z. Wu, M. Fang, and N. Li. 2015. Genome wide association analysis reveals new production trait genes in a male Duroc population. PLoS One. 10:e0139207. doi: https://doi.org/10.1371/journal.pone.0139207 26418247 PMC4587933

[CIT0057] Warde-Farley, D., S. L. Donaldson, O. Comes, K. Zuberi, R. Badrawi, P. Chao, M. Franz, C. Grouios, F. Kazi, C. T. Lopes, et al 2010. The GeneMANIA prediction server: biological network integration for gene prioritization and predicting gene function. Nucleic Acids Res. 38:W214–W220. doi: https://doi.org/10.1093/nar/gkq537 20576703 PMC2896186

[CIT0058] Willer, C. J., Y. Li, and G. R. Abecasis. 2010. METAL: fast and efficient meta-analysis of genomewide association scans. Bioinformatics. 26:2190–2191. doi: https://doi.org/10.1093/bioinformatics/btq340 20616382 PMC2922887

[CIT0059] Xu, Y., X. Qi, M. Hu, R. Lin, Y. Hou, Z. Wang, H. Zhou, Y. Zhao, Y. Luan, S. Zhao, et al 2018. Transcriptome analysis of adipose tissue indicates that the cAMP signaling pathway affects the feed efficiency of pigs. Genes. 9:336. doi: https://doi.org/10.3390/genes9070336 29973485 PMC6070815

[CIT0060] Zeggini, E., and J. P. A. Ioannidis. 2009. Meta-analysis in genome-wide association studies. Future Med. Pharmacogenimics. 10:191–201. doi: https://doi.org/10.2217/14622416.10.2.191 PMC2695132 19207020

