# Supplementary material for: A meta-analysis of genome-wide association studies to identify candidate genes associated with feed efficiency traits in pigs
Source: J Anim Sci. 2025 Jan 23;103:skaf010. doi: 10.1093/jas/skaf010 (PMC11833465; doi:10.1093/jas/skaf010)
Supplement: skaf010_suppl_Supplementary_Material [file skaf010_suppl_supplementary_material.docx]

#### **SUPPLEMENTARY MATERIAL**

| **Table S1**. Single Nucleotide Polymorphisms (SNPs) previously associated with feed conversion ratio (FCR) and residual feed intake in pigs. | | | | | | | |
| --- | --- | --- | --- | --- | --- | --- | --- |
| Author | SNPchip | Trait | Number of observations | SNP | CHR | Genomic position | P-value |
| Belous et al. (2019) | Illumina Porcine SNP60 BeadChip | FCR | 715 | rs322861732 | 2 | 28,160,205 | 0.00000319 |
| Belous et al. (2019) | Illumina Porcine SNP60 BeadChip | FCR | 715 | rs332408271 | 2 | 27,690,713 | 0.000002 |
| Belous et al. (2019) | Illumina Porcine SNP60 BeadChip | FCR | 715 | rs341847211 | 2 | 28,107,543 | 0.00000319 |
| Belous et al. (2019) | Illumina Porcine SNP60 BeadChip | FCR | 715 | rs342013568 | 2 | 26,610,361 | 0.00000257 |
| Belous et al. (2019) | Illumina Porcine SNP60 BeadChip | FCR | 715 | rs81317829 | 2 | 27,916,909 | 0.00000432 |
| Belous et al. (2019) | Illumina Porcine SNP60 BeadChip | FCR | 715 | rs81330735 | 2 | 26,792,270 | 0.00000284 |
| Belous et al. (2019) | Illumina Porcine SNP60 BeadChip | FCR | 715 | rs81356589 | 2 | 27,133,981 | 0.00000392 |
| Belous et al. (2019) | Illumina Porcine SNP60 BeadChip | FCR | 715 | rs81356686 | 2 | 28,291,546 | 0.00000201 |
| Belous et al. (2019) | Illumina Porcine SNP60 BeadChip | FCR | 715 | rs81356693 | 2 | 28,404,691 | 0.00000328 |
| Belous et al. (2019) | Illumina Porcine SNP60 BeadChip | FCR | 715 | rs81356722 | 2 | 28,370,069 | 0.00000172 |
| Belous et al. (2019) | Illumina Porcine SNP60 BeadChip | FCR | 715 | rs81363704 | 2 | 119,547,373 | 0.00000373 |
| Belous et al. (2019) | Illumina Porcine SNP60 BeadChip | FCR | 715 | rs334331384 | 3 | 4,404,919 | 0.00000183 |
| Belous et al. (2019) | Illumina Porcine SNP60 BeadChip | FCR | 715 | rs335445826 | 3 | 8,959,142 | 0.00000456 |
| Belous et al. (2019) | Illumina Porcine SNP60 BeadChip | FCR | 715 | rs81327212 | 3 | 4,544,334 | 0.00000103 |
| Belous et al. (2019) | Illumina Porcine SNP60 BeadChip | FCR | 715 | rs81369032 | 3 | 8,142,551 | 0.00000224 |
| Belous et al. (2019) | Illumina Porcine SNP60 BeadChip | FCR | 715 | rs81375722 | 3 | 110,100,507 | 0.000000414 |
| Belous et al. (2019) | Illumina Porcine SNP60 BeadChip | FCR | 715 | rs332555715 | 4 | 128,397,522 | 0.0000044 |
| Belous et al. (2019) | Illumina Porcine SNP60 BeadChip | FCR | 715 | rs329632313 | 6 | 85,493,952 | 0.00000722 |
| Belous et al. (2019) | Illumina Porcine SNP60 BeadChip | FCR | 715 | rs333263885 | 6 | 85,385,407 | 0.00000722 |
| Belous et al. (2019) | Illumina Porcine SNP60 BeadChip | FCR | 715 | rs336409671 | 6 | 86,345,973 | 0.00000157 |
| Belous et al. (2019) | Illumina Porcine SNP60 BeadChip | FCR | 715 | rs81343499 | 6 | 86,401,903 | 0.00000157 |
| Belous et al. (2019) | Illumina Porcine SNP60 BeadChip | FCR | 715 | rs81389224 | 6 | 86,333,941 | 0.00000117 |
| Belous et al. (2019) | Illumina Porcine SNP60 BeadChip | FCR | 715 | rs81389246 | 6 | 86,282,384 | 0.00000157 |
| Belous et al. (2019) | Illumina Porcine SNP60 BeadChip | FCR | 715 | rs81476027 | 6 | 86,273,741 | 0.00000987 |
| Belous et al. (2019) | Illumina Porcine SNP60 BeadChip | FCR | 715 | rs80887364 | 7 | 102,851,360 | 0.00000798 |
| Belous et al. (2019) | Illumina Porcine SNP60 BeadChip | FCR | 715 | rs344609508 | 12 | 16,010,788 | 0.00000647 |
| Belous et al. (2019) | Illumina Porcine SNP60 BeadChip | FCR | 715 | rs80859153 | 15 | 62,211,674 | 0.0000102 |
| Belous et al. (2019) | Illumina Porcine SNP60 BeadChip | FCR | 715 | rs80938723 | 15 | 63,541,092 | 0.00000918 |
| Belous et al. (2019) | Illumina Porcine SNP60 BeadChip | FCR | 715 | rs80956594 | 15 | 62,234,985 | 0.00000918 |
| Belous et al. (2019) | Illumina Porcine SNP60 BeadChip | FCR | 715 | rs81291577 | 15 | 30,615,152 | 0.00000369 |
| Belous et al. (2019) | Illumina Porcine SNP60 BeadChip | FCR | 715 | rs81301069 | 15 | 43,810,887 | 0.00000254 |
| Ding et al. (2017) | Porcine SNP60 Beadchip llumina | FCR | 338 | rs81228724 | 12 | 18,089,508 | 0.0000164 |
| Ding et al. (2017) | Porcine SNP60 Beadchip llumina | FCR | 338 | rs81244225 | 12 | 17,862,124 | 0.0000144 |
| Ding et al. (2017) | Porcine SNP60 Beadchip llumina | FCR | 338 | rs81319708 | 12 | 18,094,410 | 0.0000164 |
| Ding et al. (2018) | Geneseek Porcine 50K SNP Chip | FCR | 1,008 | rs80795431 | 1 | 172,286,374 | 0.000026 |
| Ding et al. (2018) | Geneseek Porcine 50K SNP Chip | FCR | 1,008 | rs80853064 | 1 | 173,801,989 | 0.0000161 |
| Ding et al. (2018) | Geneseek Porcine 50K SNP Chip | FCR | 1,008 | rs80872918 | 1 | 173,241,334 | 0.0000174 |
| Ding et al. (2018) | Geneseek Porcine 50K SNP Chip | FCR | 1,008 | rs80887408 | 1 | 173,838,073 | 0.0000161 |
| Ding et al. (2018) | Geneseek Porcine 50K SNP Chip | FCR | 1,008 | rs80897170 | 1 | 173,749,334 | 0.0000163 |
| Ding et al. (2018) | Geneseek Porcine 50K SNP Chip | FCR | 1,008 | rs80919991 | 1 | 173,146,830 | 0.00000872 |
| Ding et al. (2018) | Geneseek Porcine 50K SNP Chip | FCR | 1,008 | rs80939464 | 1 | 174,008,560 | 0.0000221 |
| Ding et al. (2018) | Geneseek Porcine 50K SNP Chip | FCR | 1,008 | rs80948504 | 1 | 174,142,217 | 0.0000161 |
| Ding et al. (2018) | Geneseek Porcine 50K SNP Chip | FCR | 1,008 | rs80976610 | 1 | 173,858,164 | 0.0000161 |
| Ding et al. (2018) | Geneseek Porcine 50K SNP Chip | FCR | 1,008 | rs81349630 | 1 | 172,353,082 | 0.000026 |
| Ding et al. (2018) | Geneseek Porcine 50K SNP Chip | FCR | 1,008 | rs81349654 | 1 | 173,699,271 | 0.0000161 |
| Ding et al. (2018) | Geneseek Porcine 50K SNP Chip | FCR | 1,008 | rs81314854 | 3 | 92,925,143 | 0.0000189 |
| Ding et al. (2018) | Geneseek Porcine 50K SNP Chip | FCR | 1,008 | rs80889405 | 11 | 73,310,954 | 0.00000228 |
| Ding et al. (2018) | Geneseek Porcine 50K SNP Chip | FCR | 1,008 | rs80997384 | 17 | 17,867,190 | 0.0000299 |
| Fu et al. (2020) | PorcineSNP50 BeadChip | FCR | 296 | rs80883237 | 7 | 45,856,658 | 0.000411 |
| Fu et al. (2020) | PorcineSNP50 BeadChip | FCR | 296 | rs80895086 | 7 | 45,828,581 | 0.000562 |
| Fu et al. (2020) | PorcineSNP50 BeadChip | FCR | 296 | rs345794390 | 13 | 3,655,526 | 0.00035 |
| Fu et al. (2020) | PorcineSNP50 BeadChip | FCR | 296 | rs80812508 | 14 | 40,548,521 | 0.000708 |
| Fu et al. (2020) | PorcineSNP50 BeadChip | FCR | 296 | rs80904397 | 14 | 40,507,969 | 0.000682 |
| Fu et al. (2020) | PorcineSNP50 BeadChip | FCR | 296 | rs330850570 | 17 | 1,636,763 | 0.000213 |
| Fu et al. (2020) | PorcineSNP50 BeadChip | FCR | 296 | rs335751907 | 17 | 1,152,272 | 0.000078 |
| Fu et al. (2020) | PorcineSNP50 BeadChip | FCR | 296 | rs342832896 | 17 | 1,935,638 | 0.000223 |
| Fu et al. (2020) | PorcineSNP50 BeadChip | FCR | 296 | rs344116455 | 17 | 1,956,407 | 0.00017 |
| Fu et al. (2020) | PorcineSNP50 BeadChip | FCR | 296 | rs698819984 | 17 | 2,346,395 | 0.00068 |
| Fu et al. (2020) | PorcineSNP50 BeadChip | FCR | 296 | rs80821766 | 17 | 1,703,513 | 0.000117 |
| Fu et al. (2020) | PorcineSNP50 BeadChip | FCR | 296 | rs337939758 | 18 | 20,961,153 | 0.000205 |
| Fu et al. (2020) | PorcineSNP50 BeadChip | FCR | 296 | rs81245790 | 18 | 20,484,602 | 0.000631 |
| Fu et al. (2020) | PorcineSNP50 BeadChip | FCR | 296 | rs81467760 | 18 | 23,538,749 | 0.000733 |
| Fu et al. (2020) | PorcineSNP50 BeadChip | FCR | 296 | rs334464676 | 17 | 2,686,699 | 0.000536 |
| Horodyska et al. (2017) | PorcineSNP60 BeadChip | FCR | 952 | rs80892627 | 1 | 75,408,202 | 0.000239883 |
| Horodyska et al. (2017) | PorcineSNP60 BeadChip | FCR | 952 | rs80847745 | 4 | 81,479,518 | 0.000000145 |
| Horodyska et al. (2017) | PorcineSNP60 BeadChip | FCR | 952 | rs80848071 | 4 | 81,493,481 | 0.000000123 |
| Horodyska et al. (2017) | PorcineSNP60 BeadChip | FCR | 952 | rs80903322 | 4 | 79,331,939 | 0.0000005495 |
| Horodyska et al. (2017) | PorcineSNP60 BeadChip | FCR | 952 | rs80996243 | 4 | 80,728,673 | 0.00000001096 |
| Horodyska et al. (2017) | PorcineSNP60 BeadChip | FCR | 952 | rs81382168 | 4 | 79,493,443 | 0.00000033884 |
| Horodyska et al. (2017) | PorcineSNP60 BeadChip | FCR | 952 | rs81389211 | 6 | 84,705,728 | 0.00000036308 |
| Horodyska et al. (2017) | PorcineSNP60 BeadChip | FCR | 952 | rs81389383 | 6 | 86,804,762 | 0.00000037154 |
| Horodyska et al. (2017) | PorcineSNP60 BeadChip | FCR | 952 | rs80896554 | 15 | 132,562,815 | 0.00000003981 |
| Horodyska et al. (2017) | PorcineSNP60 BeadChip | FCR | 952 | rs81326442 | 15 | 134,782,929 | 0.00000053703 |
| Li et al. (2020) | CAU50K | FCR | 485 | rs81383976 | 5 | 43,879,295 | 0.000000736 |
| Li et al. (2020) | CAU50K | FCR | 485 | rs81383984 | 5 | 44,464,360 | 0.0000267 |
| Miao et al. (2021) | Illumina PorcineSNP60 BeadChip | FCR | 3672 | rs342109728 | 3 | 79,049,546 | 0.00000102 |
| Miao et al. (2021) | Illumina PorcineSNP60 BeadChip | FCR | 3672 | rs322002490 | 5 | 38,864,890 | 0.0000013 |
| Miao et al. (2021) | Illumina PorcineSNP60 BeadChip | FCR | 3672 | rs323754097 | 5 | 36,346,640 | 0.000000975 |
| Miao et al. (2021) | Illumina PorcineSNP60 BeadChip | FCR | 3672 | rs324415971 | 5 | 37,876,819 | 0.00000102 |
| Miao et al. (2021) | Illumina PorcineSNP60 BeadChip | FCR | 3672 | rs332237334 | 5 | 33,842,149 | 0.000000479 |
| Miao et al. (2021) | Illumina PorcineSNP60 BeadChip | FCR | 3672 | rs334378193 | 5 | 38,853,109 | 0.00000143 |
| Miao et al. (2021) | Illumina PorcineSNP60 BeadChip | FCR | 3672 | rs339913443 | 5 | 35,929,672 | 0.000000661 |
| Miao et al. (2021) | Illumina PorcineSNP60 BeadChip | FCR | 3672 | rs345043801 | 5 | 33,871,482 | 0.000000603 |
| Miao et al. (2021) | Illumina PorcineSNP60 BeadChip | FCR | 3672 | rs80785563 | 5 | 33,946,621 | 0.000000603 |
| Miao et al. (2021) | Illumina PorcineSNP60 BeadChip | FCR | 3672 | rs80786392 | 5 | 33,912,700 | 0.000000451 |
| Miao et al. (2021) | Illumina PorcineSNP60 BeadChip | FCR | 3672 | rs80811321 | 5 | 30,820,701 | 0.00000126 |
| Miao et al. (2021) | Illumina PorcineSNP60 BeadChip | FCR | 3672 | rs80816650 | 5 | 34,256,405 | 0.000000492 |
| Miao et al. (2021) | Illumina PorcineSNP60 BeadChip | FCR | 3672 | rs80827728 | 5 | 38,080,702 | 0.0000011 |
| Miao et al. (2021) | Illumina PorcineSNP60 BeadChip | FCR | 3672 | rs80832154 | 5 | 34,967,936 | 0.000000701 |
| Miao et al. (2021) | Illumina PorcineSNP60 BeadChip | FCR | 3672 | rs80833936 | 5 | 34,933,883 | 0.000000701 |
| Miao et al. (2021) | Illumina PorcineSNP60 BeadChip | FCR | 3672 | rs80835055 | 5 | 34,189,654 | 0.0000007 |
| Miao et al. (2021) | Illumina PorcineSNP60 BeadChip | FCR | 3672 | rs80837106 | 5 | 33,991,092 | 0.000000451 |
| Miao et al. (2021) | Illumina PorcineSNP60 BeadChip | FCR | 3672 | rs80841312 | 5 | 33,897,913 | 0.000000439 |
| Miao et al. (2021) | Illumina PorcineSNP60 BeadChip | FCR | 3672 | rs80841410 | 5 | 34,303,955 | 0.000000492 |
| Miao et al. (2021) | Illumina PorcineSNP60 BeadChip | FCR | 3672 | rs80845463 | 5 | 34,022,700 | 0.000000451 |
| Miao et al. (2021) | Illumina PorcineSNP60 BeadChip | FCR | 3672 | rs80850598 | 5 | 34,747,588 | 0.000000493 |
| Miao et al. (2021) | Illumina PorcineSNP60 BeadChip | FCR | 3672 | rs80881700 | 5 | 37,422,475 | 0.00000121 |
| Miao et al. (2021) | Illumina PorcineSNP60 BeadChip | FCR | 3672 | rs80892229 | 5 | 34,769,398 | 0.000000744 |
| Miao et al. (2021) | Illumina PorcineSNP60 BeadChip | FCR | 3672 | rs80896133 | 5 | 37,322,781 | 0.00000124 |
| Miao et al. (2021) | Illumina PorcineSNP60 BeadChip | FCR | 3672 | rs80911792 | 5 | 41,078,366 | 0.00000142 |
| Miao et al. (2021) | Illumina PorcineSNP60 BeadChip | FCR | 3672 | rs80938383 | 5 | 40,804,197 | 0.00000146 |
| Miao et al. (2021) | Illumina PorcineSNP60 BeadChip | FCR | 3672 | rs80942282 | 5 | 35,093,967 | 0.000000741 |
| Miao et al. (2021) | Illumina PorcineSNP60 BeadChip | FCR | 3672 | rs80943228 | 5 | 35,016,164 | 0.000000701 |
| Miao et al. (2021) | Illumina PorcineSNP60 BeadChip | FCR | 3672 | rs80953078 | 5 | 38,079,753 | 0.00000102 |
| Miao et al. (2021) | Illumina PorcineSNP60 BeadChip | FCR | 3672 | rs80955114 | 5 | 34,990,669 | 0.000000701 |
| Miao et al. (2021) | Illumina PorcineSNP60 BeadChip | FCR | 3672 | rs80957248 | 5 | 37,397,149 | 0.00000121 |
| Miao et al. (2021) | Illumina PorcineSNP60 BeadChip | FCR | 3672 | rs80957355 | 5 | 38,185,708 | 0.0000011 |
| Miao et al. (2021) | Illumina PorcineSNP60 BeadChip | FCR | 3672 | rs80958876 | 5 | 40,434,314 | 0.0000015 |
| Miao et al. (2021) | Illumina PorcineSNP60 BeadChip | FCR | 3672 | rs80964107 | 5 | 38,960,519 | 0.0000013 |
| Miao et al. (2021) | Illumina PorcineSNP60 BeadChip | FCR | 3672 | rs80964888 | 5 | 33,934,311 | 0.000000472 |
| Miao et al. (2021) | Illumina PorcineSNP60 BeadChip | FCR | 3672 | rs80989003 | 5 | 33,669,222 | 0.000000439 |
| Miao et al. (2021) | Illumina PorcineSNP60 BeadChip | FCR | 3672 | rs80989707 | 5 | 33,970,407 | 0.000000635 |
| Miao et al. (2021) | Illumina PorcineSNP60 BeadChip | FCR | 3672 | rs80994480 | 5 | 35,036,218 | 0.000000701 |
| Miao et al. (2021) | Illumina PorcineSNP60 BeadChip | FCR | 3672 | rs81000718 | 5 | 34,677,764 | 0.000000701 |
| Miao et al. (2021) | Illumina PorcineSNP60 BeadChip | FCR | 3672 | rs81212454 | 5 | 38,794,710 | 0.00000146 |
| Miao et al. (2021) | Illumina PorcineSNP60 BeadChip | FCR | 3672 | rs81230832 | 5 | 38,880,761 | 0.0000013 |
| Miao et al. (2021) | Illumina PorcineSNP60 BeadChip | FCR | 3672 | rs81287625 | 5 | 34,177,721 | 0.000000525 |
| Miao et al. (2021) | Illumina PorcineSNP60 BeadChip | FCR | 3672 | rs81303224 | 5 | 38,033,697 | 0.00000105 |
| Miao et al. (2021) | Illumina PorcineSNP60 BeadChip | FCR | 3672 | rs81319635 | 5 | 34,221,053 | 0.000000552 |
| Miao et al. (2021) | Illumina PorcineSNP60 BeadChip | FCR | 3672 | rs81323542 | 5 | 44,096,325 | 0.00000137 |
| Miao et al. (2021) | Illumina PorcineSNP60 BeadChip | FCR | 3672 | rs81331039 | 5 | 44,127,767 | 0.00000149 |
| Miao et al. (2021) | Illumina PorcineSNP60 BeadChip | FCR | 3672 | rs81331835 | 5 | 44,121,830 | 0.00000149 |
| Miao et al. (2021) | Illumina PorcineSNP60 BeadChip | FCR | 3672 | rs81344478 | 5 | 33,838,344 | 0.000000479 |
| Miao et al. (2021) | Illumina PorcineSNP60 BeadChip | FCR | 3672 | rs81383574 | 5 | 30,276,591 | 0.000000879 |
| Miao et al. (2021) | Illumina PorcineSNP60 BeadChip | FCR | 3672 | rs81383707 | 5 | 34,122,773 | 0.000000454 |
| Miao et al. (2021) | Illumina PorcineSNP60 BeadChip | FCR | 3672 | rs81383732 | 5 | 35,634,440 | 0.00000101 |
| Miao et al. (2021) | Illumina PorcineSNP60 BeadChip | FCR | 3672 | rs81383754 | 5 | 36,197,319 | 0.00000103 |
| Miao et al. (2021) | Illumina PorcineSNP60 BeadChip | FCR | 3672 | rs81383786 | 5 | 36,903,934 | 0.00000126 |
| Miao et al. (2021) | Illumina PorcineSNP60 BeadChip | FCR | 3672 | rs81383847 | 5 | 37,889,695 | 0.00000102 |
| Miao et al. (2021) | Illumina PorcineSNP60 BeadChip | FCR | 3672 | rs81383849 | 5 | 37,914,858 | 0.00000102 |
| Miao et al. (2021) | Illumina PorcineSNP60 BeadChip | FCR | 3672 | rs81383856 | 5 | 37,997,338 | 0.00000102 |
| Miao et al. (2021) | Illumina PorcineSNP60 BeadChip | FCR | 3672 | rs81383857 | 5 | 38,009,941 | 0.00000146 |
| Miao et al. (2021) | Illumina PorcineSNP60 BeadChip | FCR | 3672 | rs81383866 | 5 | 38,110,593 | 0.00000141 |
| Miao et al. (2021) | Illumina PorcineSNP60 BeadChip | FCR | 3672 | rs81383891 | 5 | 38,815,027 | 0.00000158 |
| Miao et al. (2021) | Illumina PorcineSNP60 BeadChip | FCR | 3672 | rs329844461 | 15 | 16,281,234 | 0.0000011 |
| Reyer et al. (2017) | Porcine SNP60 Beadchips | FCR | 846 | rs81270901 | 6 | 104,183,387 | 0.000002570 |
| Reyer et al. (2017) | Porcine SNP60 Beadchips | FCR | 846 | rs81310751 | 6 | 101,311,106 | 0.000000028 |
| Reyer et al. (2017) | Porcine SNP60 Beadchips | FCR | 846 | rs81344722 | 6 | 111,400,426 | 0.000000083 |
| Reyer et al. (2017) | Porcine SNP60 Beadchips | FCR | 846 | rs81389928 | 6 | 95,114,962 | 0.000020893 |
| Reyer et al. (2017) | Porcine SNP60 Beadchips | FCR | 846 | rs80838208 | 7 | 117,217,668 | 0.000075858 |
| Reyer et al. (2017) | Porcine SNP60 Beadchips | FCR | 846 | rs81266686 | 9 | 111,867,248 | 0.000008511 |
| Reyer et al. (2017) | Porcine SNP60 Beadchips | FCR | 846 | rs81332374 | 9 | 135,187,034 | 0.003162278 |
| Reyer et al. (2017) | Porcine SNP60 Beadchips | FCR | 846 | rs81346296 | 9 | 109,802,987 | 0.000003802 |
| Reyer et al. (2017) | Porcine SNP60 Beadchips | FCR | 846 | rs81416088 | 9 | 115,687,227 | 0.000002570 |
| Reyer et al. (2017) | Porcine SNP60 Beadchips | FCR | 846 | rs80878204 | 11 | 24,524,578 | 0.000001549 |
| Reyer et al. (2017) | Porcine SNP60 Beadchips | FCR | 846 | rs80844227 | 14 | 98,677,304 | 0.002089296 |
| Reyer et al. (2017) | Porcine SNP60 Beadchips | FCR | 846 | rs81453027 | 15 | 50,628,285 | 0.0000676083 |
| Sahana et al. (2013) | Illumina Porcine SNP60 BeadChip | FCR | 3071 | rs80807306 | 14 | 112,353,593 | 0.0000000214 |
| Sahana et al. (2013) | Illumina Porcine SNP60 BeadChip | FCR | 3071 | rs80840893 | 14 | 111,758,839 | 0.0000002037 |
| Sahana et al. (2013) | Illumina Porcine SNP60 BeadChip | FCR | 3071 | rs80853351 | 14 | 110,994,066 | 0.0000008204 |
| Sahana et al. (2013) | Illumina Porcine SNP60 BeadChip | FCR | 3071 | rs80882443 | 14 | 111,671,437 | 0.0000001972 |
| Sahana et al. (2013) | Illumina Porcine SNP60 BeadChip | FCR | 3071 | rs80898194 | 14 | 112,271,963 | 0.0000000161 |
| Sahana et al. (2013) | Illumina Porcine SNP60 BeadChip | FCR | 3071 | rs80938302 | 14 | 113,642,045 | 0.0000000927 |
| Sahana et al. (2013) | Illumina Porcine SNP60 BeadChip | FCR | 3071 | rs80955217 | 14 | 112,646,857 | 0.0000000090 |
| Sahana et al. (2013) | Illumina Porcine SNP60 BeadChip | FCR | 3071 | rs80976779 | 14 | 111,896,310 | 0.0000002716 |
| Sahana et al. (2013) | Illumina Porcine SNP60 BeadChip | FCR | 3071 | rs80987116 | 14 | 112,381,190 | 0.0000000129 |
| Wang et al. (2015) | Illumina Porcine SNP60 BeadChip | FCR | 796 | rs336083023 | 4 | 96,632,994 | 0.044 |
| Wang et al. (2015) | Illumina Porcine SNP60 BeadChip | FCR | 796 | rs340527699 | 4 | 102,036,416 | 0.0313 |
| Wang et al. (2015) | Illumina Porcine SNP60 BeadChip | FCR | 796 | rs80934608 | 15 | 112,225,927 | 0.0451 |
| Wang et al. (2015) | Illumina Porcine SNP60 BeadChip | FCR | 796 | rs81454344 | 15 | 112,052,254 | 0.0451 |
| Bai et al. (2017) | Illumina PorcineSNP60v2 BeadChip | RFI | 217 | rs80782607 | 1 | 23,143,307 | 0.000000038 |
| Bai et al. (2017) | Illumina PorcineSNP60v2 BeadChip | RFI | 217 | rs80928833 | 1 | 23,100,880 | 0.000000380 |
| Bai et al. (2017) | Illumina PorcineSNP60v2 BeadChip | RFI | 217 | rs81367118 | 2 | 146,148,111 | 0.000000365 |
| Bai et al. (2017) | Illumina PorcineSNP60v2 BeadChip | RFI | 217 | rs81367093 | 2 | 146,286,826 | 0.000000976 |
| Bai et al. (2017) | Illumina PorcineSNP60v2 BeadChip | RFI | 217 | rs81303936 | 10 | 64,664,567 | 0.000000744 |
| Bai et al. (2017) | Illumina PorcineSNP60v2 BeadChip | RFI | 217 | rs81314967 | 10 | 66,885,948 | 0.000000243 |
| Bai et al. (2017) | Illumina PorcineSNP60v2 BeadChip | RFI | 217 | rs81225502 | 10 | 66,864,936 | 0.000000149 |
| Bai et al. (2017) | Illumina PorcineSNP60v2 BeadChip | RFI | 217 | rs81335643 | 12 | 52,538,083 | 0.000000962 |
| Bai et al. (2017) | Illumina PorcineSNP60v2 BeadChip | RFI | 217 | rs80890689 | 13 | 194,871,653.0 | 0.000000711 |
| Bai et al. (2017) | Illumina PorcineSNP60v2 BeadChip | RFI | 217 | rs81270180 | 13 | 46,617,497 | 0.000000147 |
| Bai et al. (2017) | Illumina PorcineSNP60v2 BeadChip | RFI | 217 | rs81243930 | 13 | 46,647,842 | 0.000000328 |
| Bai et al. (2017) | Illumina PorcineSNP60v2 BeadChip | RFI | 217 | rs80810051 | 15 | 1,770,319 | 0.000000460 |
| Ding et al. (2018) | Geneseek Porcine 50K SNP Chip | RFI | 1008 | rs81349630 | 1 | 172,353,082 | 0.00000163 |
| Ding et al. (2018) | Geneseek Porcine 50K SNP Chip | RFI | 1008 | rs80976610 | 1 | 173,858,164 | 0.00000121 |
| Ding et al. (2018) | Geneseek Porcine 50K SNP Chip | RFI | 1008 | rs80919991 | 1 | 173,146,830 | 0.00000065 |
| Ding et al. (2018) | Geneseek Porcine 50K SNP Chip | RFI | 1008 | rs80853064 | 1 | 173,801,989 | 0.00000121 |
| Ding et al. (2018) | Geneseek Porcine 50K SNP Chip | RFI | 1008 | rs81349654 | 1 | 173,699,271 | 0.00000121 |
| Ding et al. (2018) | Geneseek Porcine 50K SNP Chip | RFI | 1008 | rs80897170 | 1 | 173,749,334 | 0.00000125 |
| Ding et al. (2018) | Geneseek Porcine 50K SNP Chip | RFI | 1008 | rs80948504 | 1 | 174,142,217 | 0.00000121 |
| Ding et al. (2018) | Geneseek Porcine 50K SNP Chip | RFI | 1008 | rs343652685 | 1 | 174,156,779 | 0.00000467 |
| Ding et al. (2018) | Geneseek Porcine 50K SNP Chip | RFI | 1008 | rs80939464 | 1 | 174,008,560 | 0.00000153 |
| Ding et al. (2018) | Geneseek Porcine 50K SNP Chip | RFI | 1008 | rs80887408 | 1 | 173,838,073 | 0.00000121 |
| Ding et al. (2018) | Geneseek Porcine 50K SNP Chip | RFI | 1008 | rs80795431 | 1 | 172,286,374 | 0.00000163 |
| Ding et al. (2018) | Geneseek Porcine 50K SNP Chip | RFI | 1008 | rs80872918 | 1 | 173,241,334 | 0.00000128 |
| Ding et al. (2018) | Geneseek Porcine 50K SNP Chip | RFI | 1008 | rs344942807 | 1 | 170,064,540 | 0.00002370 |
| Ding et al. (2018) | Geneseek Porcine 50K SNP Chip | RFI | 1008 | rs337007455 | 7 | 17,415,915 | 0.00002250 |
| Do et al. (2014) | PorcineSNP60 Illumina | RFI | 596 | rs81258794 | 1 | 7,525,648 | 0.000100 |
| Do et al. (2014) | PorcineSNP60 Illumina | RFI | 596 | rs81373421 | 3 | 89,283,831 | 0.000100 |
| Do et al. (2014) | PorcineSNP60 Illumina | RFI | 596 | rs80965843 | 7 | 91,581,930 | 0.000054 |
| Do et al. (2014) | PorcineSNP60 Illumina | RFI | 596 | rs80928116 | 7 | 17,849,496 | 0.000025 |
| Do et al. (2014) | PorcineSNP60 Illumina | RFI | 596 | rs81343022 | 8 | 81,954,795 | 0.00003600 |
| Do et al. (2014) | PorcineSNP60 Illumina | RFI | 596 | rs81401867 | 8 | 82,021,406 | 0.00000780 |
| Do et al. (2014) | PorcineSNP60 Illumina | RFI | 596 | rs81401869 | 8 | 82,005,379 | 0.00002500 |
| Do et al. (2014) | PorcineSNP60 Illumina | RFI | 596 | rs81307920 | 8 | 81,966,981 | 0.00002600 |
| Do et al. (2014) | PorcineSNP60 Illumina | RFI | 596 | rs81301816 | 8 | 26,573,019 | 0.000090 |
| Do et al. (2014) | PorcineSNP60 Illumina | RFI | 596 | rs81324693 | 9 | 129,675,437 | 0.000042 |
| Do et al. (2014) | PorcineSNP60 Illumina | RFI | 596 | rs81477738 | 10 | 40,418,554 | 0.000081 |
| Do et al. (2014) | PorcineSNP60 Illumina | RFI | 596 | rs80920844 | 14 | 129,218,494 | 0.000100 |
| Do et al. (2014) | PorcineSNP60 Illumina | RFI | 596 | rs81453514 | 15 | 73,900,108 | 0.00009700 |
| Do et al. (2014) | PorcineSNP60 Illumina | RFI | 596 | rs80983703 | 15 | 74,462,028 | 0.00009100 |
| Do et al. (2014) | PorcineSNP60 Illumina | RFI | 596 | rs81455122 | 15 | 122,718,113 | 0.000027 |
| Do et al. (2014) | PorcineSNP60 Illumina | RFI | 596 | rs81266609 | 15 | 80,306,098 | 0.000084 |
| Do et al. (2014) | PorcineSNP60 Illumina | RFI | 596 | rs80909494 | 17 | 40,099,823 | 0.000110 |
| Fu et al. (2020) | PorcineSNP50 BeadChip | RFI | 296 | rs327236185 | 1 | 16,417,185 | 0.000373000 |
| Fu et al. (2020) | PorcineSNP50 BeadChip | RFI | 296 | rs327329163 | 4 | 106,658,684 | 0.000472000 |
| Fu et al. (2020) | PorcineSNP50 BeadChip | RFI | 296 | rs324132912 | 4 | 106,826,499 | 0.000389000 |
| Fu et al. (2020) | PorcineSNP50 BeadChip | RFI | 296 | rs80875559 | 5 | 94,558,952 | 0.000593000 |
| Fu et al. (2020) | PorcineSNP50 BeadChip | RFI | 296 | rs81251279 | 5 | 11,256,612 | 0.000475000 |
| Fu et al. (2020) | PorcineSNP50 BeadChip | RFI | 296 | rs81393578 | 6 | 158,658,647 | 0.000496000 |
| Fu et al. (2020) | PorcineSNP50 BeadChip | RFI | 296 | rs81402975 | 8 | 102,999,435 | 0.000560000 |
| Fu et al. (2020) | PorcineSNP50 BeadChip | RFI | 296 | rs81311244 | 8 | 102,881,678 | 0.000563000 |
| Fu et al. (2020) | PorcineSNP50 BeadChip | RFI | 296 | rs81262025 | 10 | 40,140,575 | 0.000128000 |
| Fu et al. (2020) | PorcineSNP50 BeadChip | RFI | 296 | rs81431225 | 11 | 5,379,554 | 0.000214000 |
| Fu et al. (2020) | PorcineSNP50 BeadChip | RFI | 296 | rs81430068 | 11 | 16,074,356 | 0.000169000 |
| Fu et al. (2020) | PorcineSNP50 BeadChip | RFI | 296 | rs80983830 | 11 | 6,366,025 | 0.000334000 |
| Fu et al. (2020) | PorcineSNP50 BeadChip | RFI | 296 | rs81430022 | 11 | 16,032,696 | 0.000628000 |
| Fu et al. (2020) | PorcineSNP50 BeadChip | RFI | 296 | rs81430119 | 11 | 16,080,206 | 0.000189000 |
| Fu et al. (2020) | PorcineSNP50 BeadChip | RFI | 296 | rs319450828 | 16 | 7,078,401 | 0.000280000 |
| Fu et al. (2020) | PorcineSNP50 BeadChip | RFI | 296 | rs327697767 | 2 | 133,780,841 | 0.000177000 |
| Fu et al. (2020) | PorcineSNP50 BeadChip | RFI | 296 | rs81371975 | 3 | 68,298,863 | 0.000347000 |
| Fu et al. (2020) | PorcineSNP50 BeadChip | RFI | 296 | rs340095665 | 6 | 131,840,097 | 0.000262000 |
| Fu et al. (2020) | PorcineSNP50 BeadChip | RFI | 296 | rs81403088 | 8 | 105,105,852 | 0.000290000 |
| Fu et al. (2020) | PorcineSNP50 BeadChip | RFI | 296 | rs81258024 | 9 | 102,094,613 | 0.000527000 |
| Fu et al. (2020) | PorcineSNP50 BeadChip | RFI | 296 | rs333944426 | 9 | 112,313,852 | 0.000550000 |
| Fu et al. (2020) | PorcineSNP50 BeadChip | RFI | 296 | rs341948420 | 9 | 112,285,807 | 0.000390000 |
| Fu et al. (2020) | PorcineSNP50 BeadChip | RFI | 296 | rs329448606 | 11 | 5,092,611 | 0.000476000 |
| Li et al. (2020) | CAU50K | RFI | 880 | rs320237095 | 2 | 73,217,053 | 0.000001850 |
| Li et al. (2020) | CAU50K | RFI | 880 | rs320243411 | 2 | 66,008,692 | 0.000002510 |
| Li et al. (2020) | CAU50K | RFI | 880 | rs322933932 | 2 | 66,729,446 | 0.000002510 |
| Li et al. (2020) | CAU50K | RFI | 880 | rs324255146 | 2 | 72,934,295 | 0.000029700 |
| Li et al. (2020) | CAU50K | RFI | 880 | rs329056098 | 2 | 73,818,550 | 0.000001850 |
| Li et al. (2020) | CAU50K | RFI | 880 | rs330639556 | 2 | 66,849,146 | 0.000001750 |
| Li et al. (2020) | CAU50K | RFI | 880 | rs331867151 | 2 | 71,484,107 | 0.000002860 |
| Li et al. (2020) | CAU50K | RFI | 880 | rs333725490 | 2 | 67,397,353 | 0.000013400 |
| Li et al. (2020) | CAU50K | RFI | 880 | rs335984226 | 2 | 73,428,891 | 0.000001850 |
| Li et al. (2020) | CAU50K | RFI | 880 | rs336484525 | 2 | 71,484,462 | 0.000002860 |
| Li et al. (2020) | CAU50K | RFI | 880 | rs340791819 | 2 | 72,523,054 | 0.000020600 |
| Li et al. (2020) | CAU50K | RFI | 880 | rs343447412 | 2 | 69,408,667 | 0.000025800 |
| Li et al. (2020) | CAU50K | RFI | 880 | rs344662679 | 2 | 76,061,262 | 0.000017300 |
| Li et al. (2020) | CAU50K | RFI | 880 | rs345488861 | 2 | 73,162,496 | 0.000029700 |
| Li et al. (2020) | CAU50K | RFI | 880 | rs693098203 | 2 | 73,817,678 | 0.000001850 |
| Li et al. (2020) | CAU50K | RFI | 880 | rs712612698 | 2 | 73,232,214 | 0.000029700 |
| Li et al. (2020) | CAU50K | RFI | 880 | rs792542846 | 2 | 73,363,418 | 0.000001850 |
| Li et al. (2020) | CAU50K | RFI | 880 | rs81223451 | 2 | 72,820,932 | 0.000029700 |
| Li et al. (2020) | CAU50K | RFI | 880 | rs81225998 | 2 | 73,477,969 | 0.000029700 |
| Li et al. (2020) | CAU50K | RFI | 880 | rs81272049 | 2 | 66,370,420 | 0.000028900 |
| Li et al. (2020) | CAU50K | RFI | 485 | rs345393699 | 8 | 89,446,476 | 0.000032600 |
| Li et al. (2020) | CAU50K | RFI | 880 | rs338952192 | 15 | 119,660,194 | 0.000028400 |
| Onteru et al. (2013) | Illumina PorcineSNP60 BeadChip | RFI | 1410 | rs81343873 | 3 | 75,985,820 | 0.000005300 |
| Onteru et al. (2013) | Illumina PorcineSNP60 BeadChip | RFI | 1410 | rs80836254 | 5 | 60,462,452 | 0.000000994 |
| Onteru et al. (2013) | Illumina PorcineSNP60 BeadChip | RFI | 1410 | rs81317745 | 6 | 8,535,032 | 0.000005300 |
| Onteru et al. (2013) | Illumina PorcineSNP60 BeadChip | RFI | 1410 | rs81398306 | 7 | 89,226,744 | 0.000000233 |
| Onteru et al. (2013) | Illumina PorcineSNP60 BeadChip | RFI | 1410 | rs80864749 | 7 | 7,655,911 | 0.000000092 |
| Onteru et al. (2013) | Illumina PorcineSNP60 BeadChip | RFI | 1410 | rs81001871 | 7 | 5,050,865 | 0.000002240 |
| Onteru et al. (2013) | Illumina PorcineSNP60 BeadChip | RFI | 1410 | rs80848608 | 13 | 201,881,431 | 0.000000053 |
| Onteru et al. (2013) | Illumina PorcineSNP60 BeadChip | RFI | 1410 | rs81256772 | 13 | 115,052,269 | 0.000007210 |
| CHR = chromosome | | | | | | | |
| FCR = feed conversion ratio | | | | | | | |
| RFI = residual feed intake | | | | | | | |

| **Table S2.** Number of Single Nucleotide Polymorphisms (SNPs) per chromosome | | |
| --- | --- | --- |
| CHR Number | Number of SNPs on CHR - FCR | Number of SNPs on CHR - RFI |
| SSC1 | 12 | 18 |
| SSC2 | 11 | 23 |
| SSC3 | 7 | 3 |
| SSC4 | 8 | 2 |
| SSC5 | 60 | 3 |
| SSC6 | 13 | 3 |
| SSC7 | 4 | 7 |
| SSC8 | 0 | 10 |
| SSC9 | 4 | 4 |
| SSC10 | 0 | 5 |
| SSC11 | 2 | 6 |
| SSC12 | 4 | 1 |
| SSC13 | 2 | 5 |
| SSC14 | 12 | 1 |
| SSC15 | 11 | 6 |
| SSC16 | 0 | 1 |
| SSC17 | 12 | 1 |
| SSC18 | 5 | 0 |
| CHR = chromosome | | |
| FCR = feed conversion ratio | | |
| RFI = residual feed intake | | |

| **Table S3. Single Nucleotide Polymorphisms (SNPs) detected as significant by the meta-analysis** | | | | | | | | | |
| --- | --- | --- | --- | --- | --- | --- | --- | --- | --- |
| Trait | SNP | | CHR | Genomic position | | | | P-value | |
| FCR | rs322002490 | | 5 | 38,864,890 | | | | 0.0000013 | |
| FCR | rs322861732 | | 2 | 28,160,205 | | | | 0.00000319 | |
| FCR | rs323754097 | | 5 | 36,346,640 | | | | 0.000000975 | |
| FCR | rs324415971 | | 5 | 37,876,819 | | | | 0.00000102 | |
| FCR | rs329632313 | | 6 | 85,493,952 | | | | 0.00000722 | |
| FCR | rs329844461 | | 15 | 16,281,234 | | | | 0.0000011 | |
| FCR | rs330850570 | | 17 | 1,636,763 | | | | 0.000213 | |
| FCR | rs332237334 | | 5 | 33,842,149 | | | | 0.000000479 | |
| FCR | rs332408271 | | 2 | 27,690,713 | | | | 0.000002 | |
| FCR | rs332555715 | | 4 | 128,397,522 | | | | 0.0000044 | |
| FCR | rs333263885 | | 6 | 85,385,407 | | | | 0.00000722 | |
| FCR | rs334331384 | | 3 | 4,404,919 | | | | 0.00000183 | |
| FCR | rs334378193 | | 5 | 38,853,109 | | | | 0.00000143 | |
| FCR | rs335445826 | | 3 | 8,959,142 | | | | 0.00000456 | |
| FCR | rs335751907 | | 17 | 1,152,272 | | | | 0.000078 | |
| FCR | rs336409671 | | 6 | 86,345,973 | | | | 0.00000157 | |
| FCR | rs337939758 | | 18 | 20,961,153 | | | | 0.000205 | |
| FCR | rs339913443 | | 5 | 35,929,672 | | | | 0.000000661 | |
| FCR | rs341847211 | | 2 | 28,107,543 | | | | 0.00000319 | |
| FCR | rs342013568 | | 2 | 26,610,361 | | | | 0.00000257 | |
| FCR | rs342109728 | | 3 | 79,049,546 | | | | 0.00000102 | |
| FCR | rs342832896 | | 17 | 1,935,638 | | | | 0.000223 | |
| FCR | rs344116455 | | 17 | 1,956,407 | | | | 0.00017 | |
| FCR | rs344609508 | | 12 | 16,010,788 | | | | 0.00000647 | |
| FCR | rs345043801 | | 5 | 33,871,482 | | | | 0.000000603 | |
| FCR | rs80785563 | | 5 | 33,946,621 | | | | 0.000000603 | |
| FCR | rs80786392 | | 5 | 33,912,700 | | | | 0.000000451 | |
| FCR | rs80795431 | | 1 | 172,286,374 | | | | 0.000026 | |
| FCR | rs80807306 | | 14 | 112,353,593 | | | | 2.138E-08 | |
| FCR | rs80811321 | | 5 | 30,820,701 | | | | 0.00000126 | |
| FCR | rs80816650 | | 5 | 34,256,405 | | | | 0.000000492 | |
| FCR | rs80821766 | | 17 | 1,703,513 | | | | 0.000117 | |
| FCR | rs80827728 | | 5 | 38,080,702 | | | | 0.0000011 | |
| FCR | rs80832154 | | 5 | 34,967,936 | | | | 0.000000701 | |
| FCR | rs80833936 | | 5 | 34,933,883 | | | | 0.000000701 | |
| FCR | rs80835055 | | 5 | 34,189,654 | | | | 0.0000007 | |
| FCR | rs80837106 | | 5 | 33,991,092 | | | | 0.000000451 | |
| FCR | rs80838208 | | 7 | 117,217,668 | | | | 0.00007586 | |
| FCR | rs80840893 | | 14 | 111,758,839 | | | | 2.037E-07 | |
| FCR | rs80841312 | | 5 | 33,897,913 | | | | 0.000000439 | |
| FCR | rs80841410 | | 5 | 34,303,955 | | | | 0.000000492 | |
| FCR | rs80845463 | | 5 | 34,022,700 | | | | 0.000000451 | |
| FCR | rs80847745 | | 4 | 81,479,518 | | | | 1.445E-07 | |
| FCR | rs80848071 | | 4 | 81,493,481 | | | | 0.000000123 | |
| FCR | rs80850598 | | 5 | 34,747,588 | | | | 0.000000493 | |
| FCR | rs80853064 | | 1 | 173,801,989 | | | | 0.0000161 | |
| FCR | rs80853351 | | 14 | 110,994,066 | | | | 8.204E-07 | |
| FCR | rs80859153 | | 15 | 62,211,674 | | | | 0.0000102 | |
| FCR | rs80872918 | | 1 | 173,241,334 | | | | 0.0000174 | |
| FCR | rs80878204 | | 11 | 24,524,578 | | | | 0.000001549 | |
| FCR | rs80881700 | | 5 | 37,422,475 | | | | 0.00000121 | |
| FCR | rs80882443 | | 14 | 111,671,437 | | | | 1.972E-07 | |
| FCR | rs80887364 | | 7 | 102,851,360 | | | | 0.00000798 | |
| FCR | rs80887408 | | 1 | 173,838,073 | | | | 0.0000161 | |
| FCR | rs80889405 | | 11 | 73,310,954 | | | | 0.00000228 | |
| FCR | rs80892229 | | 5 | 34,769,398 | | | | 0.000000744 | |
| FCR | rs80892627 | | 1 | 75,408,202 | | | | 0.0002399 | |
| FCR | rs80896133 | | 5 | 37,322,781 | | | | 0.00000124 | |
| FCR | rs80896554 | | 15 | 132,562,815 | | | | 3.981E-08 | |
| FCR | rs80897170 | | 1 | 173,749,334 | | | | 0.0000163 | |
| FCR | rs80898194 | | 14 | 112,271,963 | | | | 1.611E-08 | |
| FCR | rs80903322 | | 4 | 79,331,939 | | | | 5.495E-07 | |
| FCR | rs80911792 | | 5 | 41,078,366 | | | | 0.00000142 | |
| FCR | rs80919991 | | 1 | 173,146,830 | | | | 0.00000872 | |
| FCR | rs80938302 | | 14 | 113,642,045 | | | | 9.268E-08 | |
| FCR | rs80938383 | | 5 | 40,804,197 | | | | 0.00000146 | |
| FCR | rs80938723 | | 15 | 63,541,092 | | | | 0.00000918 | |
| FCR | rs80939464 | | 1 | 174,008,560 | | | | 0.0000221 | |
| FCR | rs80942282 | | 5 | 35,093,967 | | | | 0.000000741 | |
| FCR | rs80943228 | | 5 | 35,016,164 | | | | 0.000000701 | |
| FCR | rs80948504 | | 1 | 174,142,217 | | | | 0.0000161 | |
| FCR | rs80953078 | | 5 | 38,079,753 | | | | 0.00000102 | |
| FCR | rs80955114 | | 5 | 34,990,669 | | | | 0.000000701 | |
| FCR | rs80955217 | | 14 | 112,646,857 | | | | 9.036E-09 | |
| FCR | rs80956594 | | 15 | 62,234,985 | | | | 0.00000918 | |
| FCR | rs80957248 | | 5 | 37,397,149 | | | | 0.00000121 | |
| FCR | rs80957355 | | 5 | 38,185,708 | | | | 0.0000011 | |
| FCR | rs80958876 | | 5 | 40,434,314 | | | | 0.0000015 | |
| FCR | rs80964107 | | 5 | 38,960,519 | | | | 0.0000013 | |
| FCR | rs80964888 | | 5 | 33,934,311 | | | | 0.000000472 | |
| FCR | rs80976610 | | 1 | 173,858,164 | | | | 0.0000161 | |
| FCR | rs80976779 | | 14 | 111,896,310 | | | | 2.716E-07 | |
| FCR | rs80987116 | | 14 | 112,381,190 | | | | 1.285E-08 | |
| FCR | rs80989003 | | 5 | 33,669,222 | | | | 0.000000439 | |
| FCR | rs80989707 | | 5 | 33,970,407 | | | | 0.000000635 | |
| FCR | rs80994480 | | 5 | 35,036,218 | | | | 0.000000701 | |
| FCR | rs80996243 | | 4 | 80,728,673 | | | | 1.096E-08 | |
| FCR | rs80997384 | | 17 | 17,867,190 | | | | 0.0000299 | |
| FCR | rs81000718 | | 5 | 34,677,764 | | | | 0.000000701 | |
| FCR | rs81212454 | | 5 | 38,794,710 | | | | 0.00000146 | |
| FCR | rs81228724 | | 12 | 18,089,508 | | | | 0.0000164 | |
| FCR | rs81230832 | | 5 | 38,880,761 | | | | 0.0000013 | |
| FCR | rs81244225 | | 12 | 17,862,124 | | | | 0.0000144 | |
| FCR | rs81266686 | | 9 | 111,867,248 | | | | 0.000008511 | |
| FCR | rs81270901 | | 6 | 104,183,387 | | | | 0.00000257 | |
| FCR | rs81287625 | | 5 | 34,177,721 | | | | 0.000000525 | |
| FCR | rs81291577 | | 15 | 30,615,152 | | | | 0.00000369 | |
| FCR | rs81301069 | | 15 | 43,810,887 | | | | 0.00000254 | |
| FCR | rs81303224 | | 5 | 38,033,697 | | | | 0.00000105 | |
| FCR | rs81310751 | | 6 | 101,311,106 | | | | 2.818E-08 | |
| FCR | rs81314854 | | 3 | 92,925,143 | | | | 0.0000189 | |
| FCR | rs81317829 | | 2 | 27,916,909 | | | | 0.00000432 | |
| FCR | rs81319635 | | 5 | 34,221,053 | | | | 0.000000552 | |
| FCR | rs81319708 | | 12 | 18,094,410 | | | | 0.0000164 | |
| FCR | rs81323542 | | 5 | 44,096,325 | | | | 0.00000137 | |
| FCR | rs81326442 | | 15 | 134,782,929 | | | | 0.000000537 | |
| FCR | rs81327212 | | 3 | 4,544,334 | | | | 0.00000103 | |
| FCR | rs81330735 | | 2 | 26,792,270 | | | | 0.00000284 | |
| FCR | rs81331039 | | 5 | 44,127,767 | | | | 0.00000149 | |
| FCR | rs81331835 | | 5 | 44,121,830 | | | | 0.00000149 | |
| FCR | rs81343499 | | 6 | 86,401,903 | | | | 0.00000157 | |
| FCR | rs81344478 | | 5 | 33,838,344 | | | | 0.000000479 | |
| FCR | rs81344722 | | 6 | 111,400,426 | | | | 8.318E-08 | |
| FCR | rs81346296 | | 9 | 109,802,987 | | | | 0.000003802 | |
| FCR | rs81349630 | | 1 | 172,353,082 | | | | 0.000026 | |
| FCR | rs81349654 | | 1 | 173,699,271 | | | | 0.0000161 | |
| FCR | rs81356589 | | 2 | 27,133,981 | | | | 0.00000392 | |
| FCR | rs81356686 | | 2 | 28,291,546 | | | | 0.00000201 | |
| FCR | rs81356693 | | 2 | 28,404,691 | | | | 0.00000328 | |
| FCR | rs81356722 | | 2 | 28,370,069 | | | | 0.00000172 | |
| FCR | rs81363704 | | 2 | 119,547,373 | | | | 0.00000373 | |
| FCR | rs81369032 | | 3 | 8,142,551 | | | | 0.00000224 | |
| FCR | rs81375722 | | 3 | 110,100,507 | | | | 0.000000414 | |
| FCR | rs81382168 | | 4 | 79,493,443 | | | | 3.388E-07 | |
| FCR | rs81383574 | | 5 | 30,276,591 | | | | 0.000000879 | |
| FCR | rs81383707 | | 5 | 34,122,773 | | | | 0.000000454 | |
| FCR | rs81383732 | | 5 | 35,634,440 | | | | 0.00000101 | |
| FCR | rs81383754 | | 5 | 36,197,319 | | | | 0.00000103 | |
| FCR | rs81383786 | | 5 | 36,903,934 | | | | 0.00000126 | |
| FCR | rs81383847 | | 5 | 37,889,695 | | | | 0.00000102 | |
| FCR | rs81383849 | | 5 | 37,914,858 | | | | 0.00000102 | |
| FCR | rs81383856 | | 5 | 37,997,338 | | | | 0.00000102 | |
| FCR | rs81383857 | | 5 | 38,009,941 | | | | 0.00000146 | |
| FCR | rs81383866 | | 5 | 38,110,593 | | | | 0.00000141 | |
| FCR | rs81383891 | | 5 | 38,815,027 | | | | 0.00000158 | |
| FCR | rs81383976 | | 5 | 43,879,295 | | | | 0.000000736 | |
| FCR | rs81383984 | | 5 | 44,464,360 | | | | 0.0000267 | |
| FCR | rs81389211 | | 6 | 84,705,728 | | | | 3.631E-07 | |
| FCR | rs81389224 | | 6 | 86,333,941 | | | | 0.00000117 | |
| FCR | rs81389246 | | 6 | 86,282,384 | | | | 0.00000157 | |
| FCR | rs81389383 | | 6 | 86,804,762 | | | | 3.715E-07 | |
| FCR | rs81389928 | | 6 | 95,114,962 | | | | 0.00002089 | |
| FCR | rs81416088 | | 9 | 115,687,227 | | | | 0.00000257 | |
| FCR | rs81453027 | | 15 | 50,628,285 | | | | 0.00006761 | |
| FCR | rs81476027 | | 6 | 86,273,741 | | | | 0.00000987 | |
| RFI | rs319450828 | | 16 | 7,078,401 | | | | 0.00028 | |
| RFI | rs320237095 | | 2 | 73,217,053 | | | | 0.00000185 | |
| RFI | rs320243411 | | 2 | 66,008,692 | | | | 0.00000251 | |
| RFI | rs322933932 | | 2 | 66,729,446 | | | | 0.00000251 | |
| RFI | rs324132912 | | 4 | 106,826,499 | | | | 0.000389 | |
| RFI | rs324255146 | | 2 | 72,934,295 | | | | 0.0000297 | |
| RFI | rs327236185 | | 1 | 16,417,185 | | | | 0.000373 | |
| RFI | rs327329163 | | 4 | 106,658,684 | | | | 0.000472 | |
| RFI | rs327697767 | | 2 | 133,780,841 | | | | 0.000177 | |
| RFI | rs329056098 | | 2 | 73,818,550 | | | | 0.00000185 | |
| RFI | rs329448606 | | 11 | 5,092,611 | | | | 0.000476 | |
| RFI | rs330639556 | | 2 | 66,849,146 | | | | 0.00000175 | |
| RFI | rs331867151 | | 2 | 71,484,107 | | | | 0.00000286 | |
| RFI | rs333725490 | | 2 | 67,397,353 | | | | 0.0000134 | |
| RFI | rs335984226 | | 2 | 73,428,891 | | | | 0.00000185 | |
| RFI | rs336484525 | | 2 | 71,484,462 | | | | 0.00000286 | |
| RFI | rs337007455 | | 7 | 17,415,915 | | | | 0.0000225 | |
| RFI | rs338952192 | | 15 | 119,660,194 | | | | 0.0000284 | |
| RFI | rs340095665 | | 6 | 131,840,097 | | | | 0.000262 | |
| RFI | rs340791819 | | 2 | 72,523,054 | | | | 0.0000206 | |
| RFI | rs341948420 | | 9 | 112,285,807 | | | | 0.00039 | |
| RFI | rs343447412 | | 2 | 69,408,667 | | | | 0.0000258 | |
| RFI | rs343652685 | | 1 | 174,156,779 | | | | 0.00000467 | |
| RFI | rs344662679 | | 2 | 76,061,262 | | | | 0.0000173 | |
| RFI | rs344942807 | | 1 | 170,064,540 | | | | 0.0000237 | |
| RFI | rs345393699 | | 8 | 89,446,476 | | | | 0.0000326 | |
| RFI | rs345488861 | | 2 | 73,162,496 | | | | 0.0000297 | |
| RFI | rs693098203 | | 2 | 73,817,678 | | | | 0.00000185 | |
| RFI | rs712612698 | | 2 | 73,232,214 | | | | 0.0000297 | |
| RFI | rs792542846 | | 2 | 73,363,418 | | | | 0.00000185 | |
| RFI | rs80782607 | | 1 | 23,143,307 | | | | 0.000000038 | |
| RFI | rs80795431 | | 1 | 172,286,374 | | | | 0.00000163 | |
| RFI | rs80810051 | | 15 | 1,770,319 | | | | 0.00000046 | |
| RFI | rs80836254 | | 5 | 60,462,452 | | | | 0.000000994 | |
| RFI | rs80848608 | | 13 | 201,881,431 | | | | 5.25E-08 | |
| RFI | rs80853064 | | 1 | 173,801,989 | | | | 0.00000121 | |
| RFI | rs80864749 | | 7 | 7,655,911 | | | | 9.24E-08 | |
| RFI | rs80872918 | | 1 | 173,241,334 | | | | 0.00000128 | |
| RFI | rs80887408 | | 1 | 173,838,073 | | | | 0.00000121 | |
| RFI | rs80890689 | | 13 | 194,871,653 | | | | 0.000000711 | |
| RFI | rs80897170 | | 1 | 173,749,334 | | | | 0.00000125 | |
| RFI | rs80909494 | | 17 | 40,099,823 | | | | 0.00011 | |
| RFI | rs80919991 | | 1 | 173,146,830 | | | | 0.00000065 | |
| RFI | rs80920844 | | 14 | 129,218,494 | | | | 0.0001 | |
| RFI | rs80928116 | | 7 | 17,849,496 | | | | 0.000027 | |
| RFI | rs80928833 | | 1 | 23,100,880 | | | | 0.00000038 | |
| RFI | rs80939464 | | 1 | 174,008,560 | | | | 0.00000153 | |
| RFI | rs80948504 | | 1 | 174,142,217 | | | | 0.00000121 | |
| RFI | rs80965843 | | 7 | 91,581,930 | | | | 0.000054 | |
| RFI | rs80976610 | | 1 | 173,858,164 | | | | 0.00000121 | |
| RFI | rs80983703 | | 15 | 74,462,028 | | | | 0.000091 | |
| RFI | rs80983830 | | 11 | 6,366,025 | | | | 0.000334 | |
| RFI | rs81001871 | | 7 | 5,050,865 | | | | 0.00000224 | |
| RFI | rs81223451 | | 2 | 72,820,932 | | | | 0.0000297 | |
| RFI | rs81225502 | | 10 | 66,864,936 | | | | 0.000000149 | |
| RFI | rs81225998 | | 2 | 73,477,969 | | | | 0.0000297 | |
| RFI | rs81243930 | | 13 | 46,647,842 | | | | 0.000000328 | |
| RFI | rs81251279 | | 5 | 11,256,612 | | | | 0.000475 | |
| RFI | rs81256772 | | 13 | 115,052,269 | | | | 0.00000721 | |
| RFI | rs81258794 | | 1 | 7,525,648 | | | | 0.0001 | |
| RFI | rs81262025 | | 10 | 40,140,575 | | | | 0.000128 | |
| RFI | rs81266609 | | 15 | 80,306,098 | | | | 0.000084 | |
| RFI | rs81270180 | | 13 | 46,617,497 | | | | 0.000000147 | |
| RFI | rs81272049 | | 2 | 66,370,420 | | | | 0.0000289 | |
| RFI | rs81301816 | | 8 | 26,573,019 | | | | 0.00009 | |
| RFI | rs81303936 | | 10 | 64,664,567 | | | | 0.000000744 | |
| RFI | rs81307920 | | 8 | 81,966,981 | | | | 0.000026 | |
| RFI | rs81314967 | | 10 | 66,885,948 | | | | 0.000000243 | |
| RFI | rs81317745 | | 6 | 8,535,032 | | | | 0.0000053 | |
| RFI | rs81324693 | | 9 | 129,675,437 | | | | 0.000042 | |
| RFI | rs81335643 | | 12 | 52,538,083 | | | | 0.000000962 | |
| RFI | rs81343022 | | 8 | 81,954,795 | | | | 0.000036 | |
| RFI | rs81343873 | | 3 | 75,985,820 | | | | 0.0000053 | |
| RFI | rs81349630 | | 1 | 172,353,082 | | | | 0.00000163 | |
| RFI | rs81349654 | | 1 | 173,699,271 | | | | 0.00000121 | |
| RFI | rs81367093 | | 2 | 146,286,826 | | | | 0.000000976 | |
| RFI | rs81367118 | | 2 | 146,148,111 | | | | 0.000000365 | |
| RFI | rs81371975 | | 3 | 68,298,863 | | | | 0.000347 | |
| RFI | rs81373421 | | 3 | 89,283,831 | | | | 0.0001 | |
| RFI | rs81393578 | | 6 | 158,658,647 | | | | 0.000496 | |
| RFI | rs81398306 | | 7 | 89,226,744 | | | | 0.000000233 | |
| RFI | rs81401867 | | 8 | 82,021,406 | | | | 0.0000078 | |
| RFI | rs81401869 | | 8 | 82,005,379 | | | | 0.000025 | |
| RFI | rs81403088 | | 8 | 105,105,852 | | | | 0.00029 | |
| RFI | rs81430068 | | 11 | 16,074,356 | | | | 0.000169 | |
| RFI | rs81430119 | | 11 | 16,080,206 | | | | 0.000189 | |
| RFI | rs81431225 | | 11 | 5,379,554 | | | | 0.000214 | |
| RFI | rs81453514 | | 15 | 73,900,108 | | | | 0.000097 | |
| RFI | rs81455122 | | 15 | 122,718,113 | | | | 0.000027 | |
| RFI | rs81477738 | | 10 | 40,418,554 | | | | 0.000081 | |
| CHR = chromosome | | | | | | | | | |
| FCR = feed conversion ratio | | | | | | | | | |
| RFI = residual feed intake | | | | | | | | | |
| **Table S4.** Genes associated with significant Single Nucleotide Polymorphisms (SNPs) after meta-analysis | | | | | | | | | |
| Trait | SNP | Gene Ensembl | | | CHR | Gene start | Gene end | | Symbol |
| FCR | rs322002490 | ENSSSCG00000057485 | | | 5 | 38,837,753 | 38,837,855 | | U6 |
| FCR | rs322861732 | ENSSSCG00000026433 | | | 2 | 28,081,234 | 28,259,323 | | CCDC73 |
| FCR | rs323754097 | ENSSSCG00000000515 | | | 5 | 36,274,165 | 36,658,701 | | TRHDE |
| FCR | rs324415971 | - | | | 5 | - | - | | - |
| FCR | rs329632313 | ENSSSCG00000028973 | | | 6 | 85,412,134 | 85,528,715 | | PHACTR4 |
| FCR | rs329844461 | ENSSSCG00000015692 | | | 15 | 16,230,922 | 16,413,990 | | R3HDM1 |
| FCR | rs329844461 | ENSSSCG00000019503 | | | 15 | 16,273,551 | 16,273,659 | | MIR128-1 |
| FCR | rs330850570 | ENSSSCG00000006970 | | | 17 | 1,170,208 | 1,637,114 | | DLC1 |
| FCR | rs330850570 | ENSSSCG00000006972 | | | 17 | 1,610,565 | 1,611,374 | | C8orf48 |
| FCR | rs330850570 | ENSSSCG00000006970 | | | 17 | 1,170,208 | 1,637,114 | | DLC1 |
| FCR | rs330850570 | ENSSSCG00000006972 | | | 17 | 1,610,565 | 1,611,374 | | C8orf48 |
| FCR | rs332237334 | ENSSSCG00000000493 | | | 5 | 33,725,444 | 33,849,953 | | FRS2 |
| FCR | rs332408271 | ENSSSCG00000013312 | | | 2 | 27,580,129 | 27,685,843 | | HIPK3 |
| FCR | rs332555715 | ENSSSCG00000062868 | | | 4 | 128,363,200 | 128,371,734 | | NA |
| FCR | rs332555715 | ENSSSCG00000060113 | | | 4 | 128,367,448 | 128,370,694 | | NA |
| FCR | rs332555715 | ENSSSCG00000053026 | | | 4 | 128,414,054 | 128,418,482 | | NA |
| FCR | rs333263885 | ENSSSCG00000053664 | | | 6 | 85,352,219 | 85,355,380 | | NA |
| FCR | rs333263885 | ENSSSCG00000044404 | | | 6 | 85,384,080 | 85,392,291 | | MED18 |
| FCR | rs333263885 | ENSSSCG00000028973 | | | 6 | 85,412,134 | 85,528,715 | | PHACTR4 |
| FCR | rs334331384 | ENSSSCG00000052755 | | | 3 | 4,381,265 | 4,385,481 | | NA |
| FCR | rs334331384 | ENSSSCG00000031365 | | | 3 | 4,391,504 | 4,409,570 | | NA |
| FCR | rs334331384 | ENSSSCG00000008668 | | | 3 | 4,422,784 | 4,439,945 | | RBAK |
| FCR | rs334378193 | ENSSSCG00000057485 | | | 5 | 38,837,753 | 38,837,855 | | U6 |
| FCR | rs335445826 | ENSSSCG00000038901 | | | 3 | 8,931,097 | 8,937,184 | | CLDN15 |
| FCR | rs335445826 | ENSSSCG00000038938 | | | 3 | 8,937,453 | 8,946,955 | | FIS1 |
| FCR | rs335445826 | ENSSSCG00000046214 | | | 3 | 8,958,056 | 8,971,572 | | NA |
| FCR | rs335445826 | ENSSSCG00000054316 | | | 3 | 8,976,306 | 8,981,510 | | IFT22 |
| FCR | rs335751907 | ENSSSCG00000006970 | | | 17 | 1,170,208 | 1,637,114 | | DLC1 |
| FCR | rs335751907 | ENSSSCG00000006970 | | | 17 | 1,170,208 | 1,637,114 | | DLC1 |
| FCR | rs336409671 | - | | | 6 | - | - | | - |
| FCR | rs337939758 | ENSSSCG00000022865 | | | 18 | 20,951,003 | 21,758,568 | | GRM8 |
| FCR | rs337939758 | ENSSSCG00000022865 | | | 18 | 20,951,003 | 21,758,568 | | GRM8 |
| FCR | rs339913443 | ENSSSCG00000000512 | | | 5 | 35,827,473 | 35,908,385 | | TBC1D15 |
| FCR | rs341847211 | ENSSSCG00000013313 | | | 2 | 28,062,626 | 28,081,249 | | PRRG4 |
| FCR | rs341847211 | ENSSSCG00000026433 | | | 2 | 28,081,234 | 28,259,323 | | CCDC73 |
| FCR | rs342013568 | ENSSSCG00000013302 | | | 2 | 26,487,653 | 26,581,452 | | CAT |
| FCR | rs342109728 | ENSSSCG00000008372 | | | 3 | 78,907,317 | 79,257,760 | | EHBP1 |
| FCR | rs342832896 | ENSSSCG00000055908 | | | 17 | 1,867,481 | 2,012,144 | | NA |
| FCR | rs342832896 | ENSSSCG00000055908 | | | 17 | 1,867,481 | 2,012,144 | | NA |
| FCR | rs344116455 | ENSSSCG00000055908 | | | 17 | 1,867,481 | 2,012,144 | | NA |
| FCR | rs344609508 | ENSSSCG00000017299 | | | 12 | 15,969,769 | 16,052,680 | | MARCHF10 |
| FCR | rs345043801 | ENSSSCG00000000493 | | | 5 | 33,725,444 | 33,849,953 | | FRS2 |
| FCR | rs80785563 | ENSSSCG00000000499 | | | 5 | 33,904,975 | 33,956,637 | | BEST3 |
| FCR | rs80786392 | ENSSSCG00000062156 | | | 5 | 33,892,659 | 33,895,175 | | NA |
| FCR | rs80795431 | ENSSSCG00000061170 | | | 1 | 172,288,465 | 172,294,994 | | NA |
| FCR | rs80807306 | - | | | 14 | - | - | | - |
| FCR | rs80811321 | ENSSSCG00000000478 | | | 5 | 30,697,040 | 31,020,901 | | GRIP1 |
| FCR | rs80816650 | ENSSSCG00000045667 | | | 5 | 34,221,371 | 34,239,952 | | NA |
| FCR | rs80821766 | ENSSSCG00000057452 | | | 17 | 1,674,893 | 1,679,916 | | NA |
| FCR | rs80821766 | ENSSSCG00000057452 | | | 17 | 1,674,893 | 1,679,916 | | NA |
| FCR | rs80827728 | - | | | 5 | - | - | | - |
| FCR | rs80832154 | ENSSSCG00000025969 | | | 5 | 34,794,977 | 35,047,818 | | PTPRR |
| FCR | rs80833936 | ENSSSCG00000025969 | | | 5 | 34,794,977 | 35,047,818 | | PTPRR |
| FCR | rs80835055 | ENSSSCG00000023936 | | | 5 | 34,067,941 | 34,218,520 | | MYRFL |
| FCR | rs80837106 | ENSSSCG00000000500 | | | 5 | 34,003,180 | 34,053,896 | | RAB3IP |
| FCR | rs80838208 | ENSSSCG00000033807 | | | 7 | 117,242,760 | 117,290,347 | | C14orf132 |
| FCR | rs80840893 | ENSSSCG00000058770 | | | 14 | 111,733,993 | 111,737,342 | | NA |
| FCR | rs80841312 | ENSSSCG00000000496 | | | 5 | 33,858,572 | 33,873,523 | | CCT2 |
| FCR | rs80841410 | ENSSSCG00000053428 | | | 5 | 34,275,314 | 34,279,339 | | NA |
| FCR | rs80841410 | ENSSSCG00000047171 | | | 5 | 34,300,826 | 34,305,005 | | NA |
| FCR | rs80845463 | ENSSSCG00000000500 | | | 5 | 34,003,180 | 34,053,896 | | RAB3IP |
| FCR | rs80845463 | ENSSSCG00000044473 | | | 5 | 34,008,711 | 34,013,040 | | NA |
| FCR | rs80847745 | ENSSSCG00000006289 | | | 4 | 81,403,274 | 81,485,066 | | F5 |
| FCR | rs80848071 | ENSSSCG00000006289 | | | 4 | 81,403,274 | 81,485,066 | | F5 |
| FCR | rs80850598 | ENSSSCG00000000504 | | | 5 | 34,659,964 | 34,794,289 | | PTPRB |
| FCR | rs80853064 | - | | | 1 | - | - | | - |
| FCR | rs80853351 | ENSSSCG00000010543 | | | 14 | 110,955,633 | 111,037,537 | | ABCC2 |
| FCR | rs80859153 | - | | | 15 | - | - | | - |
| FCR | rs80872918 | - | | | 1 | - | - | | - |
| FCR | rs80878204 | ENSSSCG00000052062 | | | 11 | 24,468,110 | 24,505,477 | | TNFSF11 |
| FCR | rs80881700 | - | | | - | - | - | | - |
| FCR | rs80882443 | ENSSSCG00000010555 | | | 14 | 111,636,376 | 111,650,318 | | HIF1AN |
| FCR | rs80887364 | - | | | 7 | - | - | | - |
| FCR | rs80887408 | - | | | 1 | - | - | | - |
| FCR | rs80889405 | - | | | 11 | - | - | | - |
| FCR | rs80892229 | ENSSSCG00000000504 | | | 5 | 34,659,964 | 34,794,289 | | PTPRB |
| FCR | rs80892229 | ENSSSCG00000025969 | | | 5 | 34,794,977 | 35,047,818 | | PTPRR |
| FCR | rs80892627 | ENSSSCG00000004415 | | | 1 | 75,323,639 | 75,431,569 | | NA |
| FCR | rs80896133 | - | | | 5 | - | - | | - |
| FCR | rs80896554 | ENSSSCG00000022177 | | | 15 | 132,540,829 | 132,906,232 | | DIS3L2 |
| FCR | rs80897170 | - | | | 1 | - | - | | - |
| FCR | rs80898194 | ENSSSCG00000048585 | | | 14 | 112,216,732 | 112,284,224 | | NA |
| FCR | rs80903322 | - | | | 4 | - | - | | - |
| FCR | rs80911792 | - | | | 5 | - | - | | - |
| FCR | rs80919991 | ENSSSCG00000054980 | | | 1 | 173,163,633 | 173,168,800 | | NA |
| FCR | rs80938302 | ENSSSCG00000010586 | | | 14 | 113,500,842 | 113,626,003 | | SUFU |
| FCR | rs80938383 | - | | | 5 | - | - | | - |
| FCR | rs80938723 | ENSSSCG00000055550 | | | 15 | 63,509,039 | 63,515,648 | | NA |
| FCR | rs80939464 | - | | | 1 | - | - | | - |
| FCR | rs80942282 | ENSSSCG00000061118 | | | 5 | 35,103,369 | 35,109,892 | | NA |
| FCR | rs80943228 | ENSSSCG00000025969 | | | 5 | 34,794,977 | 35,047,818 | | PTPRR |
| FCR | rs80948504 | - | | | 1 | - | - | | - |
| FCR | rs80953078 | - | | | 5 | - | - | | - |
| FCR | rs80955114 | ENSSSCG00000025969 | | | 5 | 34,794,977 | 35,047,818 | | PTPRR |
| FCR | rs80955217 | ENSSSCG00000010564 | | | 14 | 112,606,196 | 112,617,319 | | POLL |
| FCR | rs80956594 | ENSSSCG00000062812 | | | 15 | 62,258,573 | 62,263,424 | | NA |
| FCR | rs80957248 | - | | | 5 | - | - | | - |
| FCR | rs80957355 | ENSSSCG00000059069 | | | 0 | 38,166,996 | 38,172,360 | | NA |
| FCR | rs80957355 | ENSSSCG00000055514 | | | 5 | 38,201,574 | 38,205,697 | | NA |
| FCR | rs80958876 | ENSSSCG00000026719 | | | 5 | 40,416,335 | 40,423,016 | | NA |
| FCR | rs80958876 | ENSSSCG00000052150 | | | 5 | 40,439,087 | 40,445,620 | | NA |
| FCR | rs80958876 | ENSSSCG00000038998 | | | 5 | 40,454,713 | 40,455,240 | | NA |
| FCR | rs80964107 | - | | | 5 | - | - | | - |
| FCR | rs80964888 | ENSSSCG00000000499 | | | 5 | 33,904,975 | 33,956,637 | | BEST3 |
| FCR | rs80976610 | ENSSSCG00000056451 | | | 1 | 173,876,267 | 173,876,400 | | NA |
| FCR | rs80976779 | ENSSSCG00000010556 | | | 14 | 111,823,308 | 111,913,969 | | PAX2 |
| FCR | rs80987116 | ENSSSCG00000010563 | | | 14 | 112,398,868 | 112,588,037 | | BTRC |
| FCR | rs80989003 | ENSSSCG00000037845 | | | 5 | 33,626,818 | 33,649,627 | | YEATS4 |
| FCR | rs80989707 | ENSSSCG00000000499 | | | 5 | 33,904,975 | 33,956,637 | | BEST3 |
| FCR | rs80994480 | ENSSSCG00000025969 | | | 5 | 34,794,977 | 35,047,818 | | PTPRR |
| FCR | rs80996243 | ENSSSCG00000060616 | | | 4 | 80,713,074 | 80,718,352 | | NA |
| FCR | rs80997384 | - | | | 17 | - | - | | - |
| FCR | rs81000718 | ENSSSCG00000052508 | | | 5 | 34,656,469 | 34,659,775 | | NA |
| FCR | rs81000718 | ENSSSCG00000000504 | | | 5 | 34,659,964 | 34,794,289 | | PTPRB |
| FCR | rs81212454 | ENSSSCG00000000519 | | | 5 | 38,766,998 | 38,815,597 | | GLIPR1 |
| FCR | rs81212454 | ENSSSCG00000033524 | | | 5 | 38,809,854 | 38,821,731 | | KRR1 |
| FCR | rs81228724 | ENSSSCG00000017328 | | | 12 | 18,037,138 | 18,082,348 | | ARHGAP27 |
| FCR | rs81230832 | - | | | 5 | - | - | | - |
| FCR | rs81244225 | ENSSSCG00000017321 | | | 12 | 17,839,555 | 17,847,014 | | LYZL6 |
| FCR | rs81266686 | ENSSSCG00000063285 | | | 9 | 111,843,860 | 111,848,763 | | NA |
| FCR | rs81270901 | ENSSSCG00000054338 | | | 6 | 104,187,469 | 104,192,996 | | NA |
| FCR | rs81287625 | ENSSSCG00000023936 | | | 5 | 34,067,941 | 34,218,520 | | MYRFL |
| FCR | rs81291577 | - | | | 15 | - | - | | - |
| FCR | rs81301069 | ENSSSCG00000015774 | | | 15 | 43,540,375 | 44,399,711 | | TENM3 |
| FCR | rs81303224 | - | | | 5 | - | - | | - |
| FCR | rs81310751 | ENSSSCG00000054462 | | | 6 | 101,290,436 | 101,294,483 | | NA |
| FCR | rs81314854 | - | | | 3 | - | - | | - |
| FCR | rs81317829 | ENSSSCG00000030326 | | | 2 | 27,855,471 | 27,899,766 | | TCP11L1 |
| FCR | rs81319635 | ENSSSCG00000023936 | | | 5 | 34,067,941 | 34,218,520 | | MYRFL |
| FCR | rs81319635 | ENSSSCG00000045667 | | | 5 | 34,221,371 | 34,239,952 | | NA |
| FCR | rs81319708 | ENSSSCG00000017328 | | | 12 | 18,037,138 | 18,082,348 | | ARHGAP27 |
| FCR | rs81323542 | ENSSSCG00000027447 | | | 5 | 43,913,693 | 44,218,049 | | TMTC1 |
| FCR | rs81326442 | - | | | 15 | - | - | | - |
| FCR | rs81327212 | ENSSSCG00000008665 | | | 3 | 4,516,510 | 4,518,684 | | NA |
| FCR | rs81330735 | ENSSSCG00000013303 | | | 2 | 26,548,852 | 26,782,172 | | ABTB2 |
| FCR | rs81331039 | ENSSSCG00000027447 | | | 5 | 43,913,693 | 44,218,049 | | TMTC1 |
| FCR | rs81331835 | ENSSSCG00000027447 | | | 5 | 43,913,693 | 44,218,049 | | TMTC1 |
| FCR | rs81343499 | - | | | 6 | - | - | | - |
| FCR | rs81344478 | ENSSSCG00000000493 | | | 5 | 33,725,444 | 33,849,953 | | FRS2 |
| FCR | rs81344478 | ENSSSCG00000000496 | | | 5 | 33,858,572 | 33,873,523 | | CCT2 |
| FCR | rs81344722 | ENSSSCG00000035965 | | | 6 | 111,407,639 | 111,422,109 | | AQP4 |
| FCR | rs81346296 | ENSSSCG00000034739 | | | 9 | 109,740,516 | 109,971,611 | | NA |
| FCR | rs81349630 | - | | | 1 | - | - | | - |
| FCR | rs81349654 | - | | | 1 | - | - | | - |
| FCR | rs81356589 | ENSSSCG00000013308 | | | 2 | 27,146,444 | 27,184,181 | | FBXO3 |
| FCR | rs81356686 | ENSSSCG00000013315 | | | 2 | 28,256,591 | 28,282,770 | | EIF3M |
| FCR | rs81356693 | ENSSSCG00000022291 | | | 2 | 28,376,901 | 28,377,003 | | U6 |
| FCR | rs81356722 | ENSSSCG00000022291 | | | 2 | 28,376,901 | 28,377,003 | | U6 |
| FCR | rs81363704 | ENSSSCG00000041901 | | | 2 | 119,479,189 | 119,652,531 | | NA |
| FCR | rs81369032 | ENSSSCG00000007645 | | | 3 | 8,114,146 | 8,119,841 | | NA |
| FCR | rs81375722 | ENSSSCG00000044640 | | | 3 | 110,068,909 | 110,102,823 | | NA |
| FCR | rs81382168 | ENSSSCG00000057091 | | | 4 | 79,396,516 | 79,575,525 | | NA |
| FCR | rs81383574 | - | | | 5 | - | - | | - |
| FCR | rs81383707 | ENSSSCG00000023936 | | | 5 | 34,067,941 | 34,218,520 | | MYRFL |
| FCR | rs81383732 | ENSSSCG00000000509 | | | 5 | 35,617,266 | 35,671,149 | | ZFC3H1 |
| FCR | rs81383754 | ENSSSCG00000046209 | | | 5 | 36,178,799 | 36,186,854 | | NA |
| FCR | rs81383754 | ENSSSCG00000054375 | | | 5 | 36,203,856 | 36,206,902 | | NA |
| FCR | rs81383754 | ENSSSCG00000058044 | | | 5 | 36,209,019 | 36,215,483 | | NA |
| FCR | rs81383754 | ENSSSCG00000059743 | | | 5 | 36,225,751 | 36,228,763 | | NA |
| FCR | rs81383786 | - | | | 5 | - | - | | - |
| FCR | rs81383847 | ENSSSCG00000053636 | | | 5 | 37,919,473 | 37,925,514 | | NA |
| FCR | rs81383849 | ENSSSCG00000053636 | | | 5 | 37,919,473 | 37,925,514 | | NA |
| FCR | rs81383856 | ENSSSCG00000054160 | | | 5 | 37,978,739 | 37,982,422 | | ATXN7L3B |
| FCR | rs81383857 | ENSSSCG00000054160 | | | 5 | 37,978,739 | 37,982,422 | | ATXN7L3B |
| FCR | rs81383866 | ENSSSCG00000055893 | | | 5 | 38,129,967 | 38,135,594 | | NA |
| FCR | rs81383891 | ENSSSCG00000000519 | | | 5 | 38,766,998 | 38,815,597 | | GLIPR1 |
| FCR | rs81383891 | ENSSSCG00000033524 | | | 5 | 38,809,854 | 38,821,731 | | KRR1 |
| FCR | rs81383891 | ENSSSCG00000057485 | | | 5 | 38,837,753 | 38,837,855 | | U6 |
| FCR | rs81383976 | ENSSSCG00000052538 | | | 5 | 43,866,554 | 43,866,838 | | NA |
| FCR | rs81383984 | ENSSSCG00000000541 | | | 5 | 44,381,129 | 44,559,374 | | FAR2 |
| FCR | rs81389211 | ENSSSCG00000003577 | | | 6 | 84,622,800 | 84,697,429 | | WASF2 |
| FCR | rs81389224 | ENSSSCG00000049560 | | | 6 | 86,304,429 | 86,305,250 | | NA |
| FCR | rs81389246 | ENSSSCG00000049560 | | | 6 | 86,304,429 | 86,305,250 | | NA |
| FCR | rs81389383 | - | | | 6 | - | - | | - |
| FCR | rs81389928 | ENSSSCG00000056202 | | | 6 | 95,070,466 | 95,114,648 | | NA |
| FCR | rs81416088 | ENSSSCG00000015488 | | | 9 | 115,550,569 | 115,659,263 | | TNFSF4 |
| FCR | rs81453027 | ENSSSCG00000015830 | | | 15 | 50,201,404 | 50,802,668 | | UNC5D |
| FCR | rs81476027 | ENSSSCG00000049560 | | | 6 | 86,304,429 | 86,305,250 | | NA |
| RFI | rs327236185 | ENSSSCG00000028867 | | | 1 | 16,400,983 | 16,446,854 | | PPIL4 |
| RFI | rs327236185 | ENSSSCG00000028867 | | | 1 | 16,400,983 | 16,446,854 | | PPIL4 |
| RFI | rs343652685 | - | | | 1 | - | - | | - |
| RFI | rs344942807 | ENSSSCG00000059984 | | | 1 | 170,034,359 | 170,039,195 | | NA |
| RFI | rs80782607 | - | | | 1 | - | - | | - |
| RFI | rs80795431 | ENSSSCG00000061170 | | | 1 | 172,288,465 | 172,294,994 | | NA |
| RFI | rs80853064 | - | | | 1 | - | - | | - |
| RFI | rs80872918 | - | | | 1 | - | - | | - |
| RFI | rs80887408 | - | | | 1 | - | - | | - |
| RFI | rs80897170 | - | | | 1 | - | - | | - |
| RFI | rs80919991 | ENSSSCG00000054980 | | | 1 | 173,163,633 | 173,168,800 | | NA |
| RFI | rs80928833 | - | | | 1 | - | - | | - |
| RFI | rs80939464 | - | | | 1 | - | - | | - |
| RFI | rs80948504 | - | | | 1 | - | - | | - |
| RFI | rs80976610 | ENSSSCG00000056451 | | | 1 | 173,876,267 | 173,876,400 | | NA |
| RFI | rs81258794 | ENSSSCG00000059431 | | | 1 | 7,527,861 | 7,528,838 | | MAS1 |
| RFI | rs81349630 | - | | | 1 | - | - | | - |
| RFI | rs81349654 | - | | | 1 | - | - | | - |
| RFI | rs320237095 | ENSSSCG00000033413 | | | 2 | 73,173,926 | 73,188,129 | | NRTN |
| RFI | rs320237095 | ENSSSCG00000040968 | | | 2 | 73,197,669 | 73,204,977 | | DUS3L |
| RFI | rs320237095 | ENSSSCG00000013527 | | | 2 | 73,207,273 | 73,265,866 | | CATSPERD |
| RFI | rs320237095 | ENSSSCG00000061996 | | | 2 | 73,248,723 | 73,248,829 | | U6 |
| RFI | rs320243411 | ENSSSCG00000013742 | | | 2 | 65,954,811 | 66,056,567 | | NFIX |
| RFI | rs320243411 | ENSSSCG00000048273 | | | 2 | 65,978,081 | 65,979,528 | | NA |
| RFI | rs322933932 | ENSSSCG00000036608 | | | 2 | 66,732,673 | 66,767,673 | | NA |
| RFI | rs324255146 | ENSSSCG00000013536 | | | 2 | 72,879,252 | 72,920,468 | | ACSBG2 |
| RFI | rs324255146 | ENSSSCG00000035403 | | | 2 | 72,950,056 | 73,051,180 | | RFX2 |
| RFI | rs327697767 | ENSSSCG00000014267 | | | 2 | 133,708,266 | 133,951,433 | | RAPGEF6 |
| RFI | rs329056098 | ENSSSCG00000024570 | | | 2 | 73,747,669 | 73,889,866 | | KDM4B |
| RFI | rs330639556 | ENSSSCG00000051836 | | | 2 | 66,824,440 | 66,827,161 | | NA |
| RFI | rs330639556 | ENSSSCG00000013715 | | | 2 | 66,829,154 | 66,853,255 | | NA |
| RFI | rs330639556 | ENSSSCG00000054319 | | | 2 | 66,855,191 | 66,861,292 | | NA |
| RFI | rs330639556 | ENSSSCG00000042811 | | | 2 | 66,859,134 | 66,861,618 | | NA |
| RFI | rs330639556 | ENSSSCG00000035663 | | | 2 | 66,872,671 | 66,890,769 | | NA |
| RFI | rs331867151 | ENSSSCG00000040377 | | | 2 | 71,454,141 | 71,468,141 | | NA |
| RFI | rs331867151 | ENSSSCG00000013573 | | | 2 | 71,470,445 | 71,472,549 | | TRAPPC5 |
| RFI | rs331867151 | ENSSSCG00000037188 | | | 2 | 71,471,417 | 71,519,421 | | PCP2 |
| RFI | rs331867151 | ENSSSCG00000033183 | | | 2 | 71,473,389 | 71,476,640 | | NA |
| RFI | rs331867151 | ENSSSCG00000013575 | | | 2 | 71,484,169 | 71,485,616 | | RETN |
| RFI | rs331867151 | ENSSSCG00000013576 | | | 2 | 71,504,569 | 71,513,422 | | STXBP2 |
| RFI | rs333725490 | ENSSSCG00000058023 | | | 2 | 67,372,510 | 67,373,355 | | OR7D2 |
| RFI | rs333725490 | ENSSSCG00000053037 | | | 2 | 67,398,730 | 67,399,668 | | NA |
| RFI | rs335984226 | ENSSSCG00000042523 | | | 2 | 73,401,028 | 73,411,574 | | TINCR |
| RFI | rs335984226 | ENSSSCG00000052254 | | | 2 | 73,427,459 | 73,432,361 | | NA |
| RFI | rs335984226 | ENSSSCG00000054618 | | | 2 | 73,433,556 | 73,439,111 | | NA |
| RFI | rs336484525 | ENSSSCG00000040377 | | | 2 | 71,454,141 | 71,468,141 | | NA |
| RFI | rs336484525 | ENSSSCG00000013573 | | | 2 | 71,470,445 | 71,472,549 | | TRAPPC5 |
| RFI | rs336484525 | ENSSSCG00000037188 | | | 2 | 71,471,417 | 71,519,421 | | PCP2 |
| RFI | rs336484525 | ENSSSCG00000033183 | | | 2 | 71,473,389 | 71,476,640 | | NA |
| RFI | rs336484525 | ENSSSCG00000013575 | | | 2 | 71,484,169 | 71,485,616 | | RETN |
| RFI | rs336484525 | ENSSSCG00000013576 | | | 2 | 71,504,569 | 71,513,422 | | STXBP2 |
| RFI | rs340791819 | ENSSSCG00000024275 | | | 2 | 72,500,149 | 72,500,463 | | NA |
| RFI | rs340791819 | ENSSSCG00000060067 | | | 2 | 72,515,064 | 72,515,366 | | NA |
| RFI | rs340791819 | ENSSSCG00000031115 | | | 2 | 72,528,400 | 72,532,951 | | CD70 |
| RFI | rs343447412 | ENSSSCG00000013639 | | | 2 | 69,365,012 | 69,399,081 | | SLC44A2 |
| RFI | rs343447412 | ENSSSCG00000013638 | | | 2 | 69,413,982 | 69,443,214 | | ILF3 |
| RFI | rs344662679 | ENSSSCG00000033949 | | | 2 | 76,039,656 | 76,083,555 | | GNG7 |
| RFI | rs345488861 | ENSSSCG00000027307 | | | 2 | 73,130,403 | 73,137,916 | | NDUFA11 |
| RFI | rs345488861 | ENSSSCG00000013530 | | | 2 | 73,152,510 | 73,171,605 | | NA |
| RFI | rs345488861 | ENSSSCG00000033413 | | | 2 | 73,173,926 | 73,188,129 | | NRTN |
| RFI | rs693098203 | ENSSSCG00000024570 | | | 2 | 73,747,669 | 73,889,866 | | KDM4B |
| RFI | rs712612698 | ENSSSCG00000040968 | | | 2 | 73,197,669 | 73,204,977 | | DUS3L |
| RFI | rs712612698 | ENSSSCG00000013527 | | | 2 | 73,207,273 | 73,265,866 | | CATSPERD |
| RFI | rs712612698 | ENSSSCG00000061996 | | | 2 | 73,248,723 | 73,248,829 | | U6 |
| RFI | rs792542846 | ENSSSCG00000013522 | | | 2 | 73,300,632 | 73,334,494 | | SAFB |
| RFI | rs792542846 | ENSSSCG00000013523 | | | 2 | 73,334,561 | 73,392,846 | | SAFB2 |
| RFI | rs81223451 | ENSSSCG00000013534 | | | 2 | 72,781,121 | 72,847,685 | | MLLT1 |
| RFI | rs81223451 | ENSSSCG00000021577 | | | 2 | 72,803,981 | 72,804,092 | | U6 |
| RFI | rs81225998 | ENSSSCG00000059446 | | | 2 | 73,481,337 | 73,486,347 | | ZNRF4 |
| RFI | rs81272049 | ENSSSCG00000013720 | | | 2 | 66,329,045 | 66,348,056 | | MAN2B1 |
| RFI | rs81272049 | ENSSSCG00000029347 | | | 2 | 66,396,671 | 66,430,405 | | ZNF791 |
| RFI | rs81367093 | - | | | 2 | - | - | | - |
| RFI | rs81367118 | ENSSSCG00000055331 | | | 2 | 146,126,813 | 146,131,979 | | NA |
| RFI | rs81343873 | ENSSSCG00000045708 | | | 3 | 75,808,213 | 76,031,291 | | NA |
| RFI | rs81371975 | ENSSSCG00000054413 | | | 3 | 68,305,451 | 68,311,385 | | NA |
| RFI | rs81373421 | - | | | 3 | - | - | | - |
| RFI | rs324132912 | ENSSSCG00000030154 | | | 4 | 106,758,836 | 106,806,863 | | RSBN1 |
| RFI | rs324132912 | ENSSSCG00000006766 | | | 4 | 106,809,391 | 106,876,846 | | PHTF1 |
| RFI | rs327329163 | ENSSSCG00000006760 | | | 4 | 106,572,755 | 106,627,418 | | HIPK1 |
| RFI | rs327329163 | ENSSSCG00000006761 | | | 4 | 106,646,011 | 106,656,452 | | DCLRE1B |
| RFI | rs327329163 | ENSSSCG00000006762 | | | 4 | 106,656,809 | 106,672,639 | | AP4B1 |
| RFI | rs327329163 | ENSSSCG00000038970 | | | 4 | 106,674,728 | 106,681,014 | | BCL2L15 |
| RFI | rs327329163 | ENSSSCG00000006764 | | | 4 | 106,686,604 | 106,758,099 | | PTPN22 |
| RFI | rs80836254 | ENSSSCG00000059349 | | | 5 | 60,443,978 | 60,448,396 | | NA |
| RFI | rs81251279 | ENSSSCG00000000141 | | | 5 | 11,213,312 | 11,229,158 | | EIF3D |
| RFI | rs81251279 | ENSSSCG00000000142 | | | 5 | 11,233,902 | 11,254,064 | | FOXRED2 |
| RFI | rs81251279 | ENSSSCG00000000144 | | | 5 | 11,264,645 | 11,283,395 | | TXN2 |
| RFI | rs340095665 | ENSSSCG00000003761 | | | 6 | 131,727,084 | 132,113,933 | | ADGRL2 |
| RFI | rs81317745 | ENSSSCG00000061282 | | | 6 | 8,499,657 | 8,503,193 | | NA |
| RFI | rs81317745 | ENSSSCG00000047139 | | | 6 | 8,513,967 | 8,520,769 | | NA |
| RFI | rs81317745 | ENSSSCG00000062307 | | | 6 | 8,515,868 | 8,520,769 | | NA |
| RFI | rs81317745 | ENSSSCG00000060996 | | | 6 | 8,525,289 | 8,530,556 | | NA |
| RFI | rs81317745 | ENSSSCG00000053839 | | | 6 | 8,527,353 | 8,530,534 | | NA |
| RFI | rs81393578 | ENSSSCG00000003846 | | | 6 | 158,537,465 | 158,763,551 | | GLIS1 |
| RFI | rs337007455 | - | | | 7 | - | - | | - |
| RFI | rs80864749 | ENSSSCG00000051558 | | | 7 | 7,574,372 | 7,640,989 | | SYCP2L |
| RFI | rs80864749 | ENSSSCG00000001045 | | | 7 | 7,651,601 | 7,774,018 | | ELOVL2 |
| RFI | rs80928116 | ENSSSCG00000053498 | | | 7 | 17,826,219 | 17,834,852 | | NA |
| RFI | rs80928116 | ENSSSCG00000056854 | | | 7 | 17,881,151 | 17,885,041 | | NA |
| RFI | rs80928116 | ENSSSCG00000053498 | | | 7 | 17,826,219 | 17,834,852 | | NA |
| RFI | rs80928116 | ENSSSCG00000056854 | | | 7 | 17,881,151 | 17,885,041 | | NA |
| RFI | rs80965843 | ENSSSCG00000002298 | | | 7 | 91,518,452 | 91,590,449 | | ZFYVE26 |
| RFI | rs80965843 | ENSSSCG00000028877 | | | 7 | 91,592,424 | 92,345,294 | | RAD51B |
| RFI | rs81001871 | ENSSSCG00000001027 | | | 7 | 5,058,817 | 5,210,270 | | BMP6 |
| RFI | rs81398306 | - | | | 7 | - | - | | - |
| RFI | rs345393699 | - | | | 8 | - | - | | - |
| RFI | rs81301816 | - | | | 8 | - | - | | - |
| RFI | rs81307920 | ENSSSCG00000040272 | | | 8 | 81,746,489 | 81,982,760 | | TTC29 |
| RFI | rs81307920 | ENSSSCG00000062405 | | | 8 | 81,987,429 | 81,988,108 | | NA |
| RFI | rs81343022 | ENSSSCG00000040272 | | | 8 | 81,746,489 | 81,982,760 | | TTC29 |
| RFI | rs81401867 | ENSSSCG00000026015 | | | 8 | 82,043,643 | 82,047,675 | | POU4F2 |
| RFI | rs81401867 | ENSSSCG00000026015 | | | 8 | 82,043,643 | 82,047,675 | | POU4F2 |
| RFI | rs81401869 | ENSSSCG00000040272 | | | 8 | 81,746,489 | 81,982,760 | | TTC29 |
| RFI | rs81401869 | ENSSSCG00000062405 | | | 8 | 81,987,429 | 81,988,108 | | NA |
| RFI | rs81403088 | ENSSSCG00000009114 | | | 8 | 105,121,529 | 105,197,141 | | PRSS12 |
| RFI | rs333944426 | ENSSSCG00000045606 | | | 9 | 112,341,044 | 112,341,993 | | NA |
| RFI | rs333944426 | ENSSSCG00000053411 | | | 9 | 112,335,391 | 112,476,074 | | NA |
| RFI | rs341948420 | - | | | 9 | - | - | | - |
| RFI | rs81324693 | ENSSSCG00000050221 | | | 9 | 129,605,449 | 129,771,888 | | NA |
| RFI | rs81324693 | ENSSSCG00000059720 | | | 9 | 129,648,871 | 129,654,529 | | NA |
| RFI | rs81324693 | ENSSSCG00000041374 | | | 9 | 129,704,083 | 129,870,858 | | NA |
| RFI | rs81225502 | ENSSSCG00000036117 | | | 10 | 66,741,291 | 66,971,746 | | NA |
| RFI | rs81262025 | - | | | 10 | - | - | | - |
| RFI | rs81303936 | ENSSSCG00000047628 | | | 10 | 64,673,925 | 64,732,648 | | NA |
| RFI | rs81314967 | ENSSSCG00000036117 | | | 10 | 66,741,291 | 66,971,746 | | NA |
| RFI | rs81477738 | - | | | 10 | - | - | | - |
| RFI | rs329448606 | ENSSSCG00000009310 | | | 11 | 5,044,329 | 5,089,766 | | POLR1D |
| RFI | rs80983830 | ENSSSCG00000025996 | | | 11 | 6,197,740 | 6,532,924 | | MTUS2 |
| RFI | rs81430068 | ENSSSCG00000040538 | | | 11 | 16,092,698 | 16,195,134 | | WDFY2 |
| RFI | rs81430119 | ENSSSCG00000040538 | | | 11 | 16,092,698 | 16,195,134 | | WDFY2 |
| RFI | rs81431225 | ENSSSCG00000009316 | | | 11 | 5,353,438 | 5,363,070 | | NA |
| RFI | rs81431225 | ENSSSCG00000009314 | | | 11 | 5,370,496 | 5,455,358 | | FLT3 |
| RFI | rs81335643 | ENSSSCG00000017932 | | | 12 | 52,464,689 | 52,506,574 | | ASGR2 |
| RFI | rs81335643 | ENSSSCG00000024926 | | | 12 | 52,536,738 | 52,544,791 | | ASGR1 |
| RFI | rs81335643 | ENSSSCG00000024285 | | | 12 | 52,550,141 | 52,575,143 | | DLG4 |
| RFI | rs80848608 | ENSSSCG00000012066 | | | 13 | 201,894,420 | 201,939,789 | | KCNJ15 |
| RFI | rs80890689 | ENSSSCG00000012034 | | | 13 | 194,774,497 | 195,005,089 | | TIAM1 |
| RFI | rs81243930 | - | | | 13 | - | - | | - |
| RFI | rs81256772 | ENSSSCG00000055040 | | | 13 | 115,049,159 | 115,055,443 | | NA |
| RFI | rs81270180 | - | | | 13 | - | - | | - |
| RFI | rs80920844 | ENSSSCG00000010683 | | | 14 | 129,113,603 | 129,347,152 | | GRK5 |
| RFI | rs338952192 | - | | | 15 | - | - | | - |
| RFI | rs80810051 | ENSSSCG00000055275 | | | 15 | 1,735,194 | 1,739,851 | | NA |
| RFI | rs80810051 | ENSSSCG00000057828 | | | 15 | 1,773,529 | 1,778,620 | | NA |
| RFI | rs80983703 | ENSSSCG00000057227 | | | 15 | 74,438,156 | 74,442,246 | | NA |
| RFI | rs80983703 | ENSSSCG00000022496 | | | 15 | 74,447,787 | 74,758,122 | | STK39 |
| RFI | rs81266609 | ENSSSCG00000044710 | | | 15 | 80,295,752 | 80,296,165 | | NA |
| RFI | rs81266609 | ENSSSCG00000055609 | | | 15 | 80,312,149 | 80,317,626 | | NA |
| RFI | rs81266609 | ENSSSCG00000036172 | | | 15 | 80,325,377 | 80,329,020 | | SP9 |
| RFI | rs81453514 | ENSSSCG00000043455 | | | 15 | 73,772,848 | 73,973,673 | | NA |
| RFI | rs81453514 | ENSSSCG00000056096 | | | 15 | 73,885,211 | 73,889,201 | | NA |
| RFI | rs81453514 | ENSSSCG00000048454 | | | 15 | 73,889,813 | 73,894,913 | | NA |
| RFI | rs81453514 | ENSSSCG00000058820 | | | 15 | 73,920,295 | 73,925,727 | | NA |
| RFI | rs81455122 | - | | | 15 | - | - | | - |
| RFI | rs319450828 | - | | | 16 | - | - | | - |
| RFI | rs80909494 | ENSSSCG00000007324 | | | 17 | 40,020,440 | 40,099,001 | | SOGA1 |
| RFI | rs80909494 | ENSSSCG00000027806 | | | 17 | 40,107,462 | 40,171,878 | | SAMHD1 |
| RFI | rs80909494 | ENSSSCG00000031690 | | | 17 | 40,109,210 | 40,120,399 | | TLDC2 |
| CHR = chromosome | | | | | | | | | |
| FCR = feed conversion ratio | | | | | | | | | |
| RFI = residual feed intake | | | | | | | | | |
| Gene start = start position of the gene in the chromosome, in basepairs | | | | | | | | | |
| Gene end = end position of the gene in the chromosome, in basepairs | | | | | | | | | |
| NA = Not available | | | | | | | | | |

| **Table S5.** Functional analysis of the genes annotated after the meta-analysis. | | | | | |
| --- | --- | --- | --- | --- | --- |
| Trait | Category | Term | Count | P-value | Genes |
| FCR | Biological Process | KW-0524 Neurogenesis | 2 | 0.093 | *MED18, PHACTR4* |
| FCR | GO: Biological Process | GO:0061386 Closure of optic fissure | 2 | **0.009** | *MED18, PHACTR4* |
| FCR | GO: Biological Process | GO:2001045 Negative regulation of integrin-mediated signaling pathway | 2 | **0.012** | *MED18, PHACTR4* |
| FCR | GO: Biological Process | GO:0033598 Mammary gland epithelial cell proliferation | 2 | **0.021** | *TNFSF11, BTRC* |
| FCR | GO: Biological Process | GO:0043085 Positive regulation of catalytic activity | 2 | **0.045** | *MED18, PHACTR4* |
| FCR | GO: Biological Process | GO:0048484 Enteric nervous system development | 2 | **0.045** | *MED18, PHACTR4* |
| FCR | GO: Biological Process | GO:0003281 Ventricular septum development | 2 | 0.054 | *FRS2, SUFU* |
| FCR | GO: Biological Process | GO:0051497 Negative regulation of stress fiber assembly | 2 | 0.060 | *DLC1, WASF2* |
| FCR | GO: Biological Process | GO:0001755 Neural crest cell migration | 2 | 0.097 | *MED18, PHACTR4* |
| FCR | GO: Biological Process | GO:0001843 Neural tube closure | 3 | **0.010** | *MED18, SUFU, PHACTR4* |
| FCR | GO: Biological Process | GO:0030036 Actin cytoskeleton organization | 4 | **0.006** | *DLC1, WASF2, MED18, PHACTR4* |
| FCR | GO: Biological Process | GO:0051726 Regulation of cell cycle | 4 | **0.009** | *MED18, YEATS4, PHACTR4, BTRC* |
| FCR | GO: Biological Process | GO:0007165 Signal transduction | 5 | **0.044** | *DLC1, TRHDE, ARHGAP27, TCP11L1, TENM3* |
| FCR | GO: Cellular Component | GO:0000178 Exosome (RNase complex) | 2 | **0.034** | *DIS3L2, ZFC3H1* |
| FCR | GO: Cellular Component | GO:0005779 Integral component of peroxisomal membrane | 2 | **0.048** | *FIS1, FAR2* |
| FCR | GO: Cellular Component | GO:0070847 Core mediator complex | 2 | 0.059 | *MED18, PHACTR4* |
| FCR | GO: Cellular Component | GO:0005778 Peroxisomal membrane | 2 | 0.070 | *FAR2, CAT* |
| FCR | GO: Cellular Component | GO:0030027 Lamellipodium | 3 | **0.045** | *WASF2, MED18, PHACTR4* |
| FCR | GO: Cellular Component | GO:0016020 Membrane | 8 | **0.012** | ENSSSCG00000056202, *DLC1*, ENSSSCG00000034739, *TRHDE, FRS2, ABCC2, TNFSF4*, ENSSSCG00000008665 |
| FCR | GO: Molecular Function | GO:0072542 Protein phosphatase activator activity | 2 | **0.023** | *MED18, PHACTR4* |
| FCR | GO: Molecular Function | GO:0008157 Protein phosphatase 1 binding | 2 | 0.062 | *MED18, PHACTR4* |
| FCR | GO: Molecular Function | GO:0005164 Tumor necrosis factor receptor binding | 2 | 0.076 | *TNFSF11, TNFSF4* |
| FCR | GO: Molecular Function | GO:0005096 GTPase activator activity | 3 | 0.086 | *DLC1, TBC1D15, ARHGAP27* |
| FCR | GO: Molecular Function | GO:0003779 Actin binding | 4 | **0.034** | ENSSSCG00000056202, *WASF2, MED18, PHACTR4* |
| FCR | Molecular Function | KW-0217 Developmental protein | 3 | 0.093 | *MED18, SUFU, PHACTR4* |
| FCR | Molecular Function | KW-9996 Developmental protein | 3 | 0.093 | *MED18, SUFU, PHACTR4* |
| FCR | Molecular Function | KW-0009 Actin-binding | 4 | **0.007** | ENSSSCG00000056202, *WASF2, MED18, PHACTR4* |
| RFI | GO: Biological Process | GO:0000724 Double-strand break repair via homologous recombination | 3 | **0.020** | *SAMHD1, RAD51B, ZFYVE26* |
| RFI | GO: Biological Process | GO:0030520 Intracellular estrogen receptor signaling pathway | 2 | **0.037** | *SAFB, POU4F2* |
| RFI | GO: Biological Process | GO:0050684 Regulation of mRNA processing | 2 | **0.041** | *SAFB, SAFB2* |
| RFI | GO: Biological Process | GO:0006357 Regulation of transcription from RNA polymerase II promoter | 8 | 0.088 | *SP9, GLIS1, SAFB, POU4F2, NFIX,* ENSSSCG00000035663, *ZNF791, RFX2* |
| RFI | GO: Cellular Component | GO:0005736 DNA-directed RNA polymerase I complex | 2 | **0.035** | *POLR1D, LOC106505238* |
| RFI | GO: Cellular Component | GO:0005666 DNA-directed RNA polymerase III complex | 2 | 0.052 | *POLR1D, LOC106505238* |
| RFI | GO: Molecular Function | GO:0030246 Carbohydrate binding | 5 | **0.001** | *ASGR1, ASGR2, MAN2B1,* ENSSSCG00000040377, *ADGRL2* |
| RFI | GO: Molecular Function | GO:0003677 DNA binding | 9 | **0.031** | *HIPK1,* ENSSSCG00000013715, *ILF3, RAD51B, NFIX, POLR1D, PHTF1, RFX2, LOC106505238* |
| RFI | GO: Molecular Function | GO:0051213 Dioxygenase activity | 2 | 0.096 | *RSBN1, KDM4B* |
| RFI | Biological Process | KW-0051 Antiviral defense | 2 | 0.073 | *SAMHD1, ILF3* |
| RFI | GO: Cellular Component | KW-9994 DNA-directed RNA polymerase | 2 | 0.067 | *POLR1D, LOC106505238* |
| RFI | GO: Cellular Component | KW-0240 DNA-directed RNA polymerase | 2 | 0.067 | *POLR1D, LOC106505238* |
| RFI | Ligand | KW-0430 Lectin | 3 | 0.090 | *ASGR1, ASGR2,* ENSSSCG00000040377 |
| FCR = feed conversion ratio | | | | | |
| RFI = residual feed intake  Bold values indicate statistical significance (p < 0.05) | | | | | |


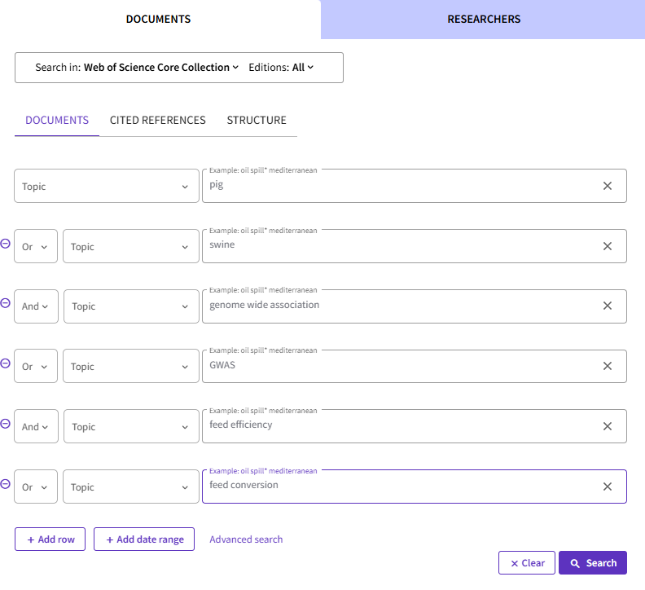

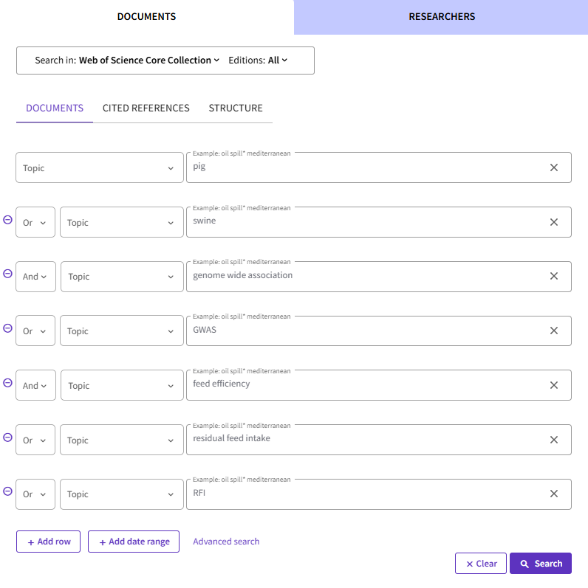

**Supplementary Figure 1. Search strings used in the Web of Science database.** Panel (a) shows the search string applied for studies related to Feed Conversion Ratio (FCR), while panel (b) displays the search string used for studies focused on Residual Feed Intake (RFI).


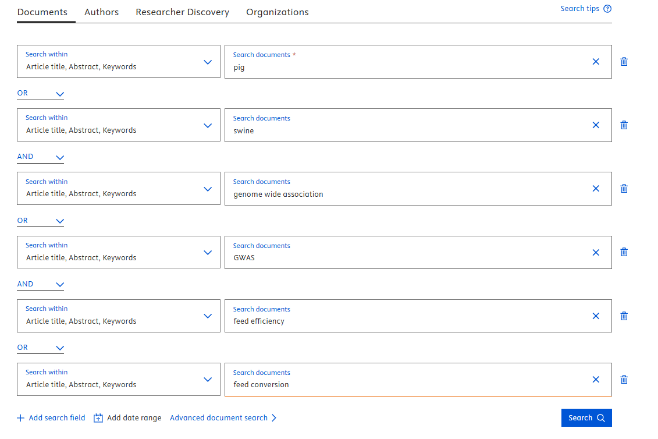

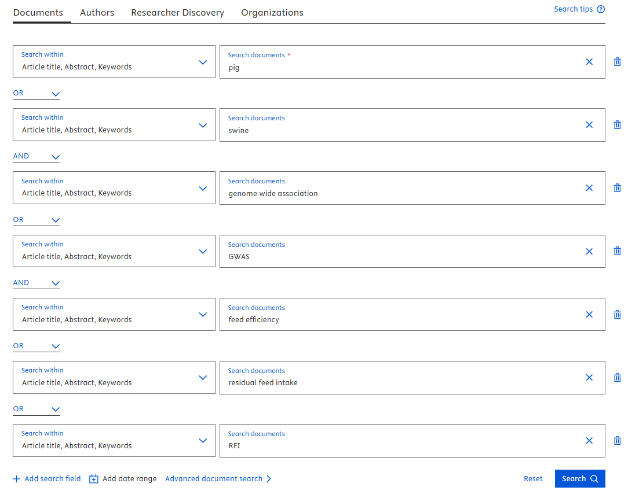


**Supplementary Figure 2. Search strings used in the Scopus database.** Panel (a) shows the search string applied for studies related to Feed Conversion Ratio (FCR), while panel (b) displays the search string used for studies focused on Residual Feed Intake (RFI).


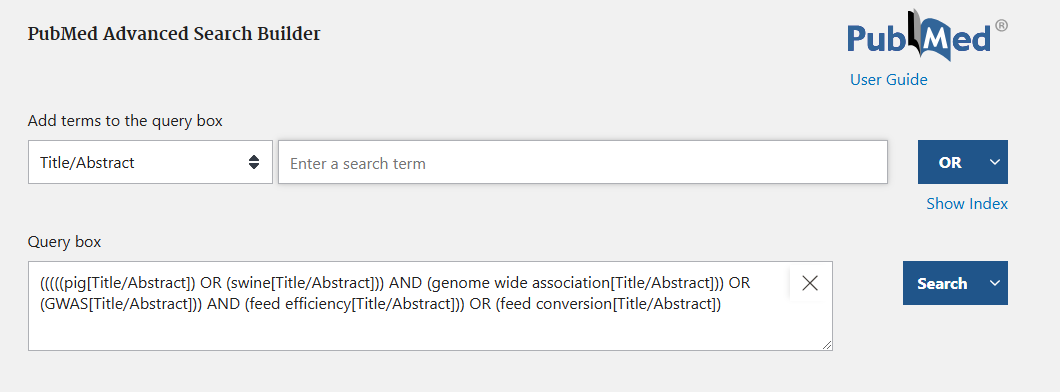

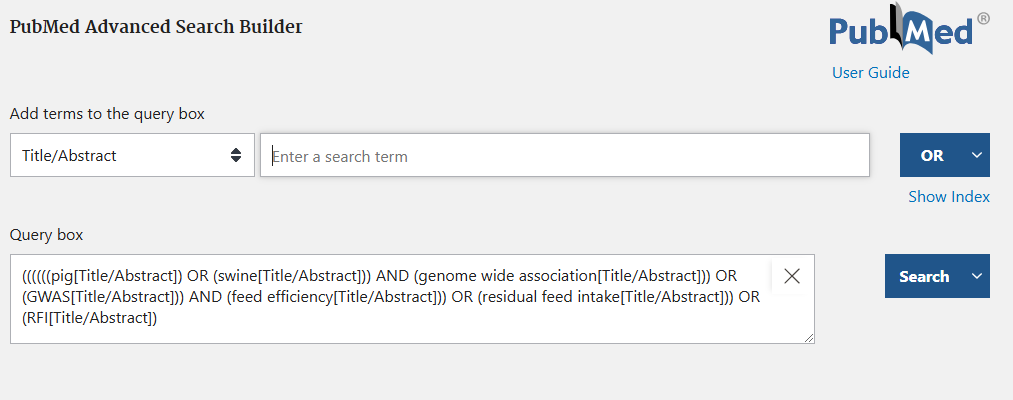


**Supplementary Figure 3. Search strings used in the Pubmed database.** Panel (a) shows the search string applied for studies related to Feed Conversion Ratio (FCR), while panel (b) displays the search string used for studies focused on Residual Feed Intake (RFI).
